# α-Hemolysin of uropathogenic *E. coli* regulates NLRP3 inflammasome activation and mitochondrial dysfunction in THP-1 macrophages

**DOI:** 10.1038/s41598-020-69501-1

**Published:** 2020-07-28

**Authors:** Vivek Verma, Parveen Kumar, Surbhi Gupta, Sonal Yadav, Rakesh Singh Dhanda, Henrik Thorlacius, Manisha Yadav

**Affiliations:** 1 Dr. B. R. Ambedkar Center for Biomedical Research, University of Delhi (North Campus), Delhi, 110007 India; 2 Department of Urology, University of Alabama At Birmingham, Hugh Kaul Genetics Building, Birmingham, AL USA; 3 Stem Cell Laboratory, Longboat Explorers AB, SMiLE Incubator, Scheelevägen 2, Lund, Sweden; 4 Department of Clinical Sciences, Section of Surgery, Malmö, Skåne University Hospital, Lund University, Malmö, Sweden

**Keywords:** Microbiology, Bacteria, Cellular microbiology, Immunology, Immune cell death, Inflammation

## Abstract

Hemolysin expressing UPEC strains have been associated with severe advanced kidney pathologies, such as cystitis and pyelonephritis, which are associated with an inflammatory response. Macrophages play an important role in regulating an inflammatory response during a urinary tract infection. We have studied the role of purified recombinant α-hemolysin in inducing inflammatory responses and cell death in macrophages. Acylation at lysine residues through HlyC is known to activate proHlyA into a fully functional pore-forming toxin, HlyA. It was observed that active α-hemolysin (HlyA) induced cleavage of caspase-1 leading to the maturation of IL-1β, while inactive α-hemolysin (proHlyA) failed to do so in THP-1 derived macrophages. HlyA also promotes deubiquitination, oligomerization, and activation of the NLRP3 inflammasome, which was found to be dependent on potassium efflux. We have also observed the co-localization of NLRP3 within mitochondria during HlyA stimulations. Moreover, blocking of potassium efflux improved the mitochondrial health in addition to a decreased inflammatory response. Our study demonstrates that HlyA stimulation caused perturbance in potassium homeostasis, which led to the mitochondrial dysfunction followed by an acute inflammatory response, resulting in cell death. However, the repletion of intracellular potassium stores could avoid HlyA induced macrophage cell death. The findings of this study will help to understand the mechanism of α-hemolysin induced inflammatory response and cell death.

## Introduction

A urinary tract infection (UTI) is one of the most common bacterial infections and is the second most common nosocomial infection^1^. Advanced stages of UTI involve uropathogenic *Escherichia coli* (UPEC) infection in the bladder (cystitis) and kidney (pyelonephritis) leading to sepsis and kidney damage. Such infections are one of the leading causes of death worldwide^1–3^. *E. coli,* generally a commensal bacteria residing inside the distal colon^4^, accounts for about 80% of the community-acquired infections of the urinary tract^5^. A UPEC mediated UTI is further complicated due to the emergence of the drug-resistant UPEC strain^6^. Therefore, it is very important to understand the pathogenesis of a UPEC mediated UTI to find an effective cure for such dreadful infections.

UPEC, which is thought to have arisen from distal gut microflora, has to pass through the challenges of the distinctly different habitats of the bladder, kidney and blood to cause an ascending UTI^7^. With time, UPEC has found ways to evade the immune system. CFT073, a virulent UPEC strain isolated from a pyelonephritis patient, was shown to induce cell death in macrophages^8^. It has been shown that macrophages are important for the recruitment of neutrophils during an experimental UTI^9^. UPEC, in comparison to its non-pathogenic commensal partners, contains extra genes coding for virulence factors which help it during pathogenesis^10^. The resulting virulence factors can be secreted toxins, such as α-hemolysin (HlyA), cytotoxic necrotizing factor 1 (CNF1), secreted auto-transporter toxin (SAT) and membrane-bound proteins (fimbriae: type 1 fim, P fim, S fim, flagellin, lipopolysaccharides and capsule)^10^.

*α*-hemolysin (HlyA) is an important toxin in the pathogenesis of UPEC, which mediates more severe forms of UTI such as cystitis and pyelonephritis^11^. An inactive α-hemolysin (non-acylated) matures within the cytoplasm to form an active toxin (acylated) through HlyC (acyltransferase) mediated fatty acylation, before being released into the extracellular environment^12,13^. HlyA has a concentration dependent effect on the epithelial cells^14^ of the kidney. At higher concentrations, it causes hemolysis of erythrocytes and other cells, thereby helping UPEC to cross mucosal barriers to cause harm to immune cells and causing the bacteria to access the nutrients of the hosts^15,16^. On the other hand, at lower concentrations it leads to apoptosis of host cells, including the immune cells (neutrophils and T lymphocytes) and kidney cells (renal cells and bladder epithelial) to ultimately cause exfoliation^17–19^. Furthermore, HlyA causes a Ca^2^^+^ imbalance in renal epithelial cells, followed by the synthesis of inflammatory cytokines IL-6 and IL-8^20^.

An inflammatory response is mediated by various pattern recognition receptors (PRRs). Inflammasomes are multi-protein complexes acting as cytosolic PRRs, which are activated during various infections^21,22^. CFT073, the UPEC strain, which kills macrophages as mentioned earlier, has been shown to activate the nod-like-receptor pyrin domain-containing 3 (NLRP3) inflammasome^8^. Contrasting reports have also shown that CFT073 blocks the activation of NLRP3 inflammasome through the virulence factor TcpC^23^. After activation of NLRP3 by various stimuli, it oligomerizes with apoptosis-associated speck-like protein containing CARD (ASC) and procaspase-1 (48 kDa) to mediate an autocatalytic cleavage of procaspase-1 into its functional form, i.e. caspase-1 (10 kDa and 20 kDa)^24,25^. A cleaved form of caspase-1, in turn, cleaves proInterleukin (proIL)-1β into its mature and secretable form that is IL-1β (17 kDa)^24,25^. Interestingly, CFT073 *hlyA* mutant could not trigger cell death and IL-1β release in mouse macrophages. However in humans, this mutant strain partially reduced the level of UPEC-triggered macrophage cell death^8^. In addition, a random transposon mutant library screen showed that HlyA had a prominent role in CFT073-triggered cell death in human macrophages^26^. Bhakdi et al. have shown that *E. coli* HlyA induces IL-1β release and cell death into monocytes^27^. Very recently, it has been shown that the UPEC α-hemolysin changes the mitochondrial dynamics in a calcium influx dependent manner in rat Sertoli cells causing mitochondrial dysfunction^28^. Also, HlyA disrupted cell membrane lead to the release of DAMP (danger associated molecular pattern), which induces a pro-inflammatory response in testicular macrophages^28^. Similarly, Schaale et al. mentioned that UPEC exhibits completely different behavior in humans and rodents^8^. Therefore, the current study was designed to study the role of uropathogenic *E. coli* α-hemolysin in the activation of NLRP3 inflammasome and mitochondrial health, with respect to cell death in human macrophages. For this, recombinant purified *E. coli* α-hemolysin was used to understand the functional effect of HlyA in macrophages derived from a human leukemic cell line, THP-1.

## Results

### HlyA promotes caspase-1 cleavage and IL-1β maturation along-with oligomerization and deubiquitination of NLRP3 in THP-1 derived macrophages

Prior to the use of recombinant hemolysin, the levels of endotoxin in our preparations of HlyA and proHlyA were checked to avoid the synergistic effect of lipopolysaccharide contamination during various stimulations. The amount of endotoxin contamination in the preparation of HlyA and proHlyA was found to be 0.0121 ± 0.0002 and 0.0119 ± 0.0001 ng/ml, respectively^29^. Recently, Schwarz et al. have reported a minimum amount of endotoxin required to elicit an immune response in human immune cells to be 0.02 ng/ml^30^. Therefore, an insignificant amount of endotoxin contamination was found in our preparations of HlyA and proHlyA, unable to produce a synergistic effect during stimulations.

**Figure 1:**
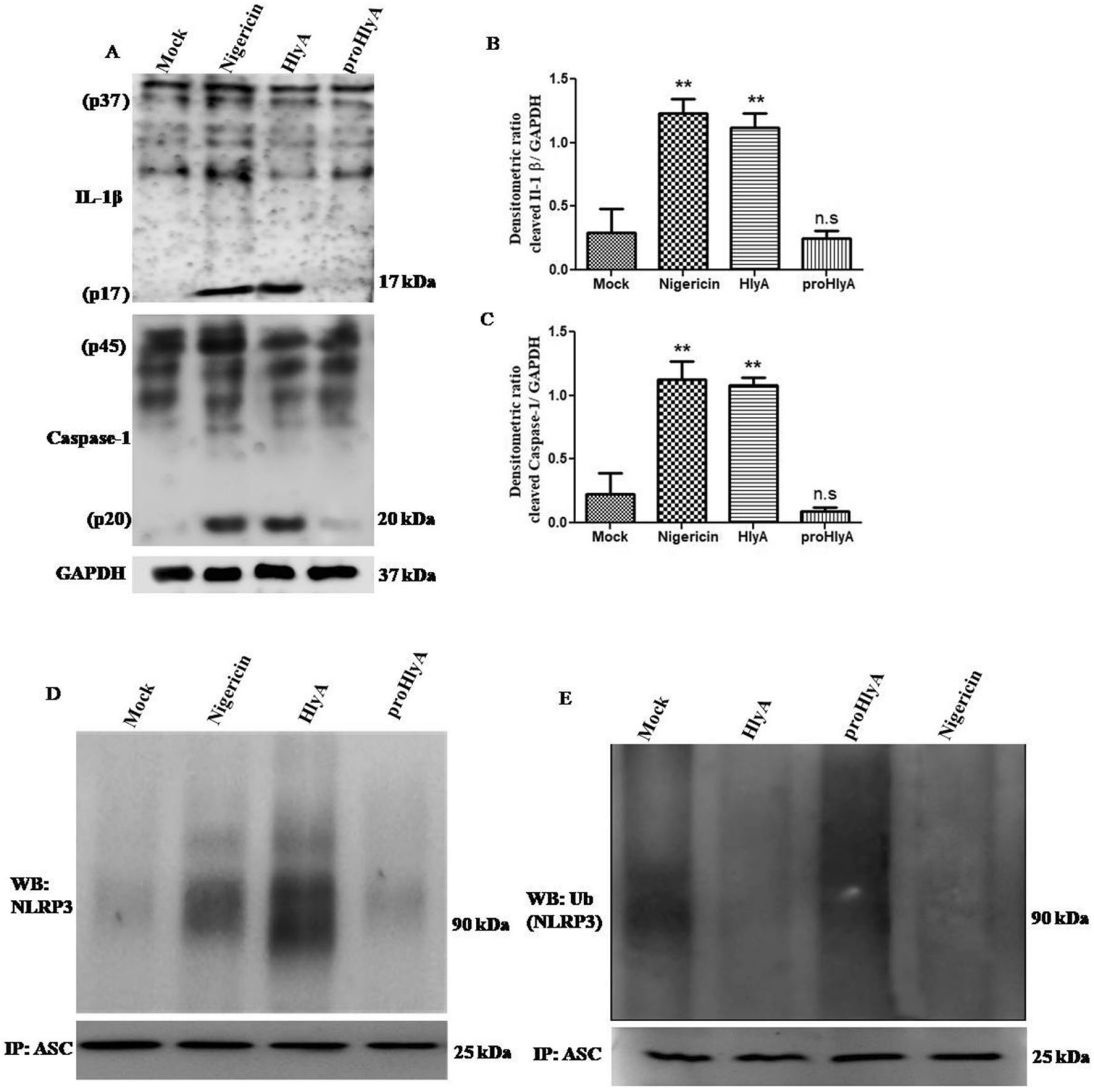
HlyA induces cleavage of IL-1β and caspase-1 with simultaneously affecting oligomerization and deubiquitination of NLRP3. THP-1m were stimulated with nigericin (30 min), HlyA and proHlyA (2 h) as indicated. Mock shows resting macrophages without any stimulation in all blots. (**A**) Immunoblots showing pro- and cleaved forms of IL-1β and caspase-1, GAPDH was used as an endogenous control. (**B**) Bar graphs showing integrated densitometric value (IDV) of cleaved IL-1β (p17) and (**C**) cleaved caspase-1 (p20). Results were expressed as Mean IDV ± SEM and analyzed by using one-way ANOVA with Bonferroni’s post test. (**D**) Co-immunoprecipitation of NLRP3 with ASC shows oligomerization of NLRP3 with ASC during various stimulations. ASC was precipitated with ASC antibody and further NLRP3 was detected by immunoblotting. ASC was also checked in input lysates through immunoblotting. (**E**) Shows endogenous ubiquitination of NLRP3 during stimulation of THP-1m with HlyA and proHlyA for 2 h. ASC was also detected in input lysates through immunoblotting. Blots are representative of three independent experiments. P value is shown as **p ≤ 0.01, n. s = non significant.

Reports suggest that *E. coli* strains carrying α-hemolysin increase the production of IL-1β from various cell types^8,26,31,32^. Similarly, we found that the levels of proIL-1β and procaspase-1 were not significantly different after stimulation and an effect was seen on the cleavage of both proteins (Fig. 1A) (Supplementary Information 1). HlyA induce significant cleavage of IL-1β (P ≤ 0.01) and caspase-1 (P ≤ 0.01) in THP-1 derived macrophages (THP-1m), whereas proHlyA failed to do so (Fig. 1A–C). Nigericin (an ionophore previously reported to promote the cleavage of IL-1β and caspase-1 through activation of NLRP3 in a potassium (K^+^) dependent manner) was used as a positive control in our experiment, because α-hemolysin of UPEC also promotes K^+^ perturbations in an intracellular mileu^33,34^.

Deubiquitination of NLRP3 is required for the activation and interaction of ASC, which is rather inhibited due to the ubiquitination of NLRP3 in resting cells (i.e. primarily macrophages)^35,36^. Therefore, we looked at the oligomerization of NLRP3 during stimulation with HlyA and proHlyA. We have observed that proHlyA failed to initiate the oligomerization of NLRP3, whereas both HlyA and nigericin induced oligomerization of NLRP3 with ASC (Fig. 1D) (Supplementary Information 1). This indicates that the pore forming property of an active α-hemolysin is important in initiating the NLRP3 inflammasome formation. Additionally, we checked the ubiquitination status of NLRP3 during HlyA and proHlyA stimulations. It was found that both HlyA and nigericin promote the deubiquitination of NLRP3, whereas proHlyA failed to do so (Fig. 1E) (Supplementary Information 1).

### HlyA promotes oligomerization and deubiquitination of NLRP3 in a potassium dependent manner

**Figure 2:**
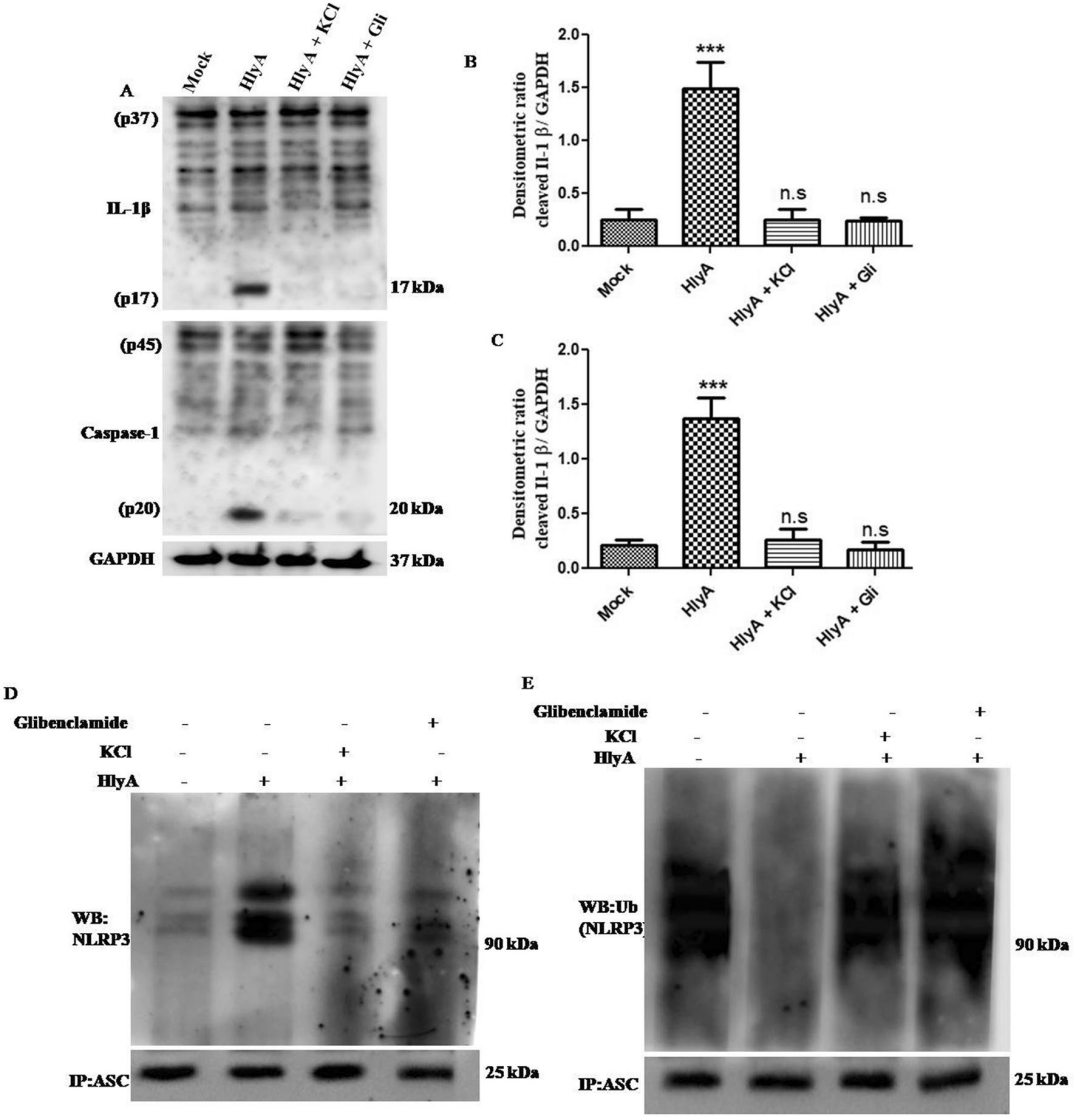
Oligomerization and deubiquitination of NLRP3 is dependent on intracellular K^+^ concentration during HlyA stimulation. THP-1m cells were treated with 140 mM of potassium chloride (KCl) and 100 μM of glibenclamide (Gli) for 30 min followed by stimulation with HlyA for 2 h. (**A**) Immunoblots showing pro and cleaved forms of IL-1β and caspase-1; GAPDH was used as endogenous control. (**B**) Bar graphs showing integrated densitometric value (IDV) of cleaved IL-1β and (**C**) cleaved caspase-1, under different HlyA stimulation, as mentioned. Results were expressed as Mean IDV ± SEM and analyzed by using one-way ANOVA with Bonferroni’s post test. (**D**) Co-immunoprecipitation with anti-ASC antibody and immunoblotting with anti-NLRP3 antibody shows oligomerization of NLRP3 with ASC during various stimulations and treatments as mentioned. ASC was also checked in input lysates by immunoblotting. (**E**) Co-immunoprecipitation with anti-ASC antibody and immunoblotting with anti-Ubiquitin antibody shows ubiquitination status of NLRP3 during various stimulations and treatments as mentioned earlier. ASC was also checked in input lysates by immunoblotting. Blots are representative of three independent experiments. P value is shown as ***p ≤ 0.001, n. s = non significant.

Kloft et al. reported that HlyA promotes intracellular potassium perturbances^34^ and that potassium oscillations in cytosol play an important role in the regulation of inflammasomes^37–39^. Thus, we hypothesized that HlyA induced inflammasomes activation might involve K^+^ perturbations. Therefore, as suggested earlier, we used a 140 mM extracellular potassium (KCl) concentration and 100 μM of glibenclamide to inhibit the effect of potassium efflux generated during action of pore-forming toxins (PFTs) on NLRP3 inflammasome activation^39^. It was observed that HlyA induced the cleavage of IL-1β and caspase-1 was inhibited in the presence of glibenclamide and high extracellular KCl (140 mM) (Fig. 2A–C) (Supplementary Information 1). However, no significant effect was seen on the pro- forms of IL-1β and caspase-1 levels. This indicates that NLRP3 inflammasome formation is inhibited due to the blockage of potassium efflux as proposed by Arlehamn et al.^39^.

The interaction between NLRP3 and ASC was also investigated by immunoprecipitation of NLRP3 inflammasome complex by anti-ASC antibody. It was found that NLRP3 oligomerization, due to HlyA stimulation, was inhibited in the presence of glibenclamide (100 μM) and higher extracellular K^+^ (140 mM KCl) **(**Fig. 2D) (Supplementary Information 1). The interaction of NLRP3 with ASC is downstream to the deubiquitination process, therefore the ubiquitination of NLRP3 during these potassium interventions was also investigated. Interestingly, we observed that ubiquitination is also a K^+^ dependent process during HlyA stimulations, because in the presence of glibenclamide and higher extracellular K^+^, deubiquitination of NLRP3 was inhibited (Fig. 2E) (Supplementary Information 1). Therefore, these results demonstrated that oligomerization and deubiquitination of NLRP3 inflammasome is dependent on intracellular K^+^ during HlyA stimulations.

### NLRP3 co-localizes in mitochondria during HlyA stimulation

**Figure 3:**
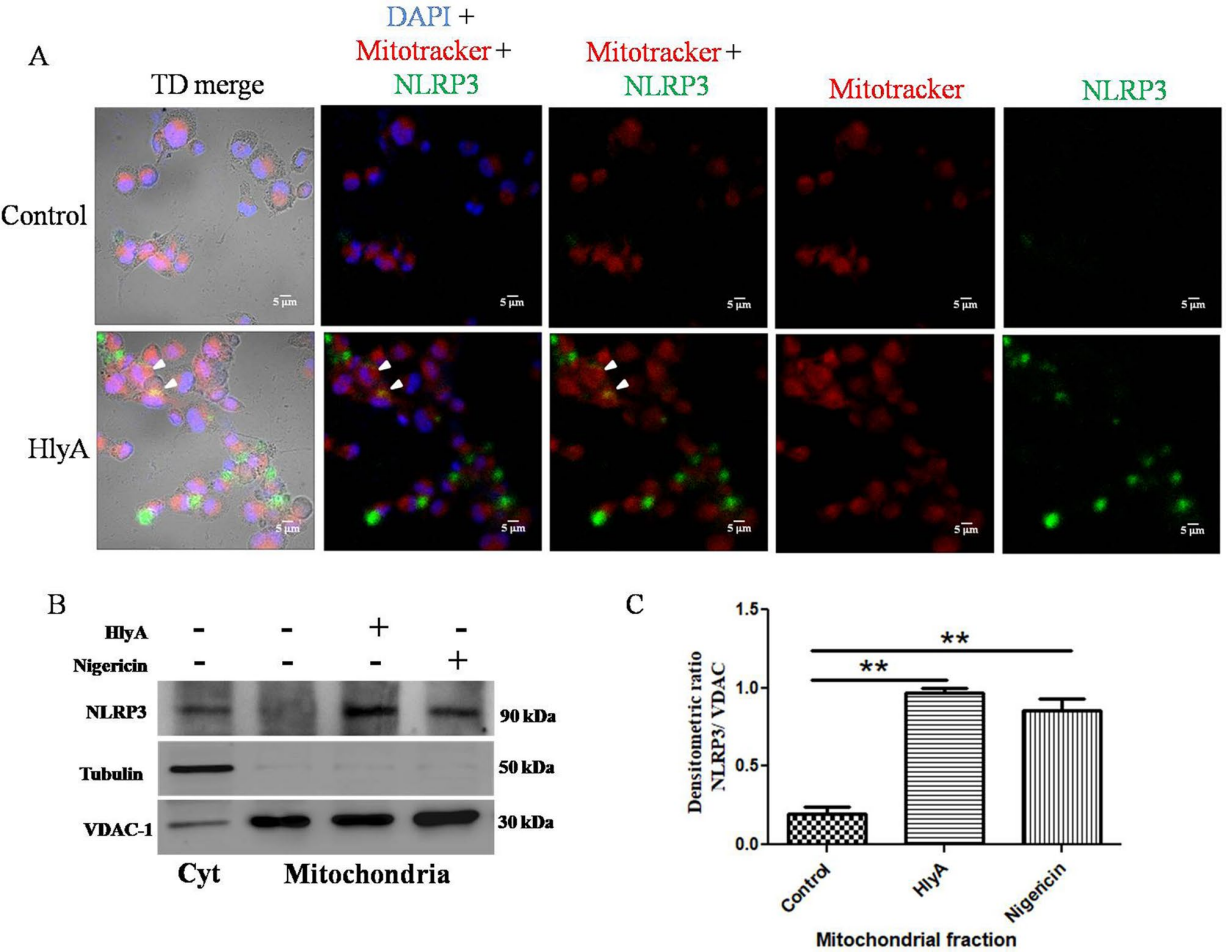
NLRP3 colocalizes in mitochondria during HlyA stimulation. (**A**) THP-1m cells were stimulated with HlyA for 2 h and then stained with Mitotracker (red) for mitochondria and DAPI (blue) for nucleus. NLRP3 was probed by anti-NLRP3 antibody and detected by secondary Alexa fluor 488 (green) and observed under ×40 objective through confocal microscopy (Scale = 5 μm). White arrows indicate the colocalization (yellow) of NLRP3 (green) with mitochondria (red). Figures are representative of 3 independent experiments. (**B**) THP-1m cells were treated with HlyA (2 h) and nigericin (30 min) as indicated and then followed for preparation of cytoplasmic extract and mitochondria isolation. Cytoplasmic extracts and mitochondrial fractions were immunoblotted for the presence of NLRP3, tubulin and VDAC1 proteins. Immunoblot shows the presence of NLRP3 in mitochondrial and cytoplasmic fractions whereas NLRP3 is present in mitochondrial fractions of HlyA stimulated cells, while absent in mitochondria from unstimulated THP-1m cells. Tubulin was immunoblotted to check the purity of mitochondrial fractions for contamination of cytoplasmic content and VDAC1 was used as a loading control for mitochondrial fractions. Blots are representative of 3 independent experiments. (**C**) Bar graph showing integrated densitometric values (IDV) of NLRP3 normalized to VDAC1 in Mitochondrial fractions. Comparisons between multiple groups were made using one-way ANOVA with Bonferroni’s post test. P value is shown as **p ≤ 0.01.

NLRP3 inflammasome plays an important role in the processing of IL-1β in cytosol to initiate a cascade of pro-inflammatory responses. Zhou et al. reported the presence of NLRP3 in mitochondria and its activation during mitochondrial dysfunction, leading to cell death in response to various stimulators, such as nigericin, monosodium urate crystals and alum^40^. We have not come across any report which shows an interaction of NLRP3 with mitochondria upon HlyA stimulation. Therefore, the localization of NLRP3 in response to HlyA stimulation was investigated. Our data shows the presence of NLRP3 in mitochondria during HlyA stimulations (arrow in Fig. 3A).

To confirm the presence of NLRP3 in mitochondria, we isolated the mitochondrial fractions from THP-1m after stimulation with HlyA. NLRP3 was found in the mitochondrial fractions isolated from HlyA- and nigericin-stimulated THP-1m (Lane 3 and 4, Fig. 3B) (Supplementary Information 1). However, NLRP3 was not detected in mitochondrial fractions of unstimulated THP-1m (Lane 2, Fig. 3B). The presence of NLRP3 was also confirmed as a positive control for NLRP3 detection in the cytoplasmic extracts of unstimulated THP-1m (Lane 1, Fig. 3B). An anti-Tubulin antibody was used to check the purity of isolated mitochondrial fractions and to eliminate the possibility of the contamination with cytoplasmic NLRP3. An anti-VDAC-1 antibody was used as a loading control for mitochondrial fractions (Fig. 3B) (Supplementary Information 1). Voltage-dependent anion channel (VDAC-1) is an ion channel in the outer membrane of the mitochondria. The densitometry analysis showed that NLRP3 in mitochondrial fractions of HlyA- and nigericin-treated cells was significantly higher (P < 0.01) than the control group (unstimulated) (Fig. 3C).

### Mitochondrial dysfunction is dependent on intracellular potassium ion concentration during HlyA stimulation of THP-1m

**Figure 4:**
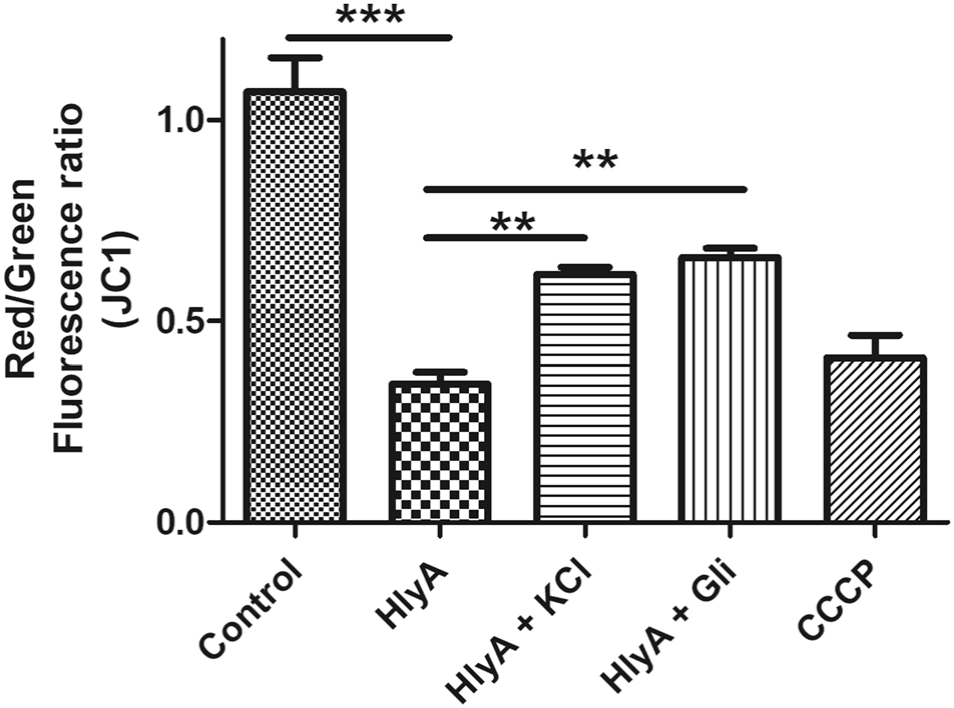
HlyA induces mitochondrial dysfunction in a potassium dependent manner. The graph shows red/green fluorescence ratio as a measurement of mitochondrial membrane potential (ΔΨm) during various stimulations of THP-1m. Cells were seeded and differentiated in 96-well clear well black plate and then stimulated with HlyA (2 h) alone or in combination with 30 min pretreatment of cells by 100 μM glibenclamide (Gli) and 140 mM KCl as indicated, followed by staining with JC1 dye for 30 min. JC1 remains as a monomer in the cytoplasm, where it gives a green fluorescence, while on its directional uptake inside the mitochondria, promoted by membrane potential, leads to the formation of JC1 aggregates, which fluoresce at red fluorescence. Fluorescence was taken at Ex 485 and Em 530 for green aggregates and Ex 488 and Em 590 for red aggregates. The graph shows decreased mitochondrial membrane potential (ΔΨm) during HlyA stimulation, whereas in the case of pretreatment with Gli and KCl along with HlyA stimulation, ΔΨm was increased. Comparisons between multiple groups were made using one-way ANOVA with Bonferroni’s post test. P value is shown as **p ≤ 0.01, ***p ≤ 0.001.

The effect of HlyA stimulation on the mitochondrial membrane potential (ΔΨm) was analyzed using the lipophilic cationic probe JC1 (Fig. 4). JC1m onomers give a green fluorescence at 529 nm. It enters inside mitochondria based on the membrane potential. Once inside the mitochondria, the JC1 form aggregates, this gives a red fluorescence at 590 nm. So ΔΨm was measured as a red/green fluorescence ratio. It was found that the HlyA stimulation significantly reduced ΔΨm as compared to the resting THP-1m (control) (p < 0.05) (Fig. 4), whereas pre-treatment of THP-1m cells with glibenclamide and high potassium containing medium significantly increased ΔΨm during HlyA stimulation. Carbonyl cyanide 3-chlorophenylhydrazone (CCCP), an oxidative phosphorylation inhibitor, was used as a control to induce depolarization of the mitochondria (Fig. 4). Therefore, it was concluded that the K^+^ concentration perturbations produced during HlyA stimulation induce mitochondrial dysfunction and repletion of potassium ion could restore the mitochondrial homeostasis.

### α-hemolysin induces oxidative stress in THP-1m, whereas repletion of potassium brought glutathione-redox status back to normal

**Figure 5:**
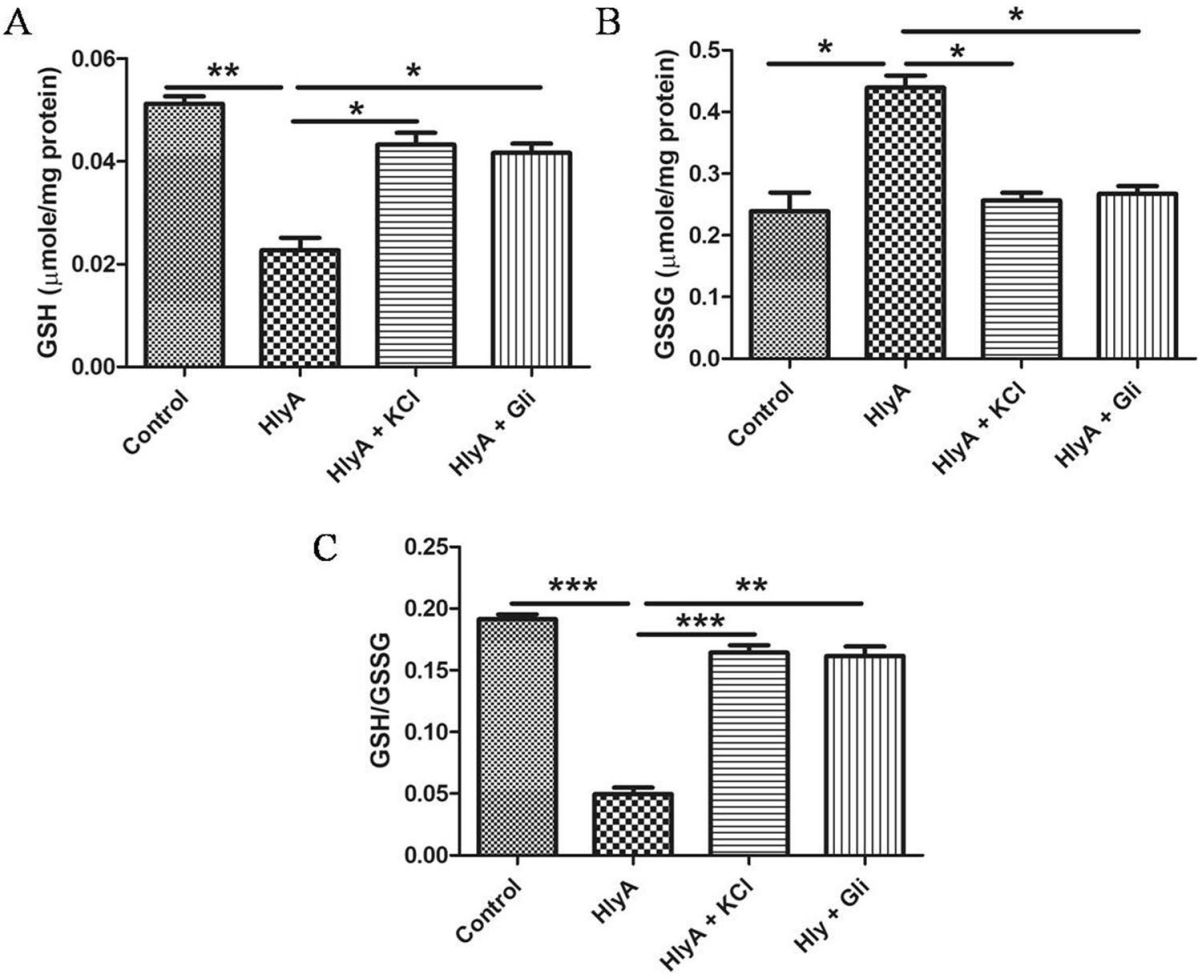
α-Hemolysin induces oxidative stress in mitochondria of THP-1m and inhibition of potassium efflux brought glutathione-redox status to normal. GSH:GSSG estimation was performed to evaluate the effect of α-hemolysin (HlyA) on THP-1m mitochondrial redox state. Additionally, the effect of glibenclamide and potassium chloride were also assessed on hemolysin induced oxidative stress in THP-1m. THP-1m pre-treated with glibenclamide (100 μM, 30 min prior to stimulation) and KCl (140 mM, 30 min prior to stimulation) were stimulated with HlyA for 2 h. Data shown is the average of three independent experiments. Comparisons between multiple groups were made using one-way ANOVA with Bonferroni’s post test. P value is shown as *p ≤ 0.05, **p ≤ 0.01, ***p ≤ 0.001.

GSH (Glutathione) is the major antioxidant defense against reactive oxygen species (ROS). The antioxidant function of GSH is determined by the redox-active thiol (-SH) of cysteine that becomes oxidized, when GSH reduces the target molecule^41^. The ratio of reduced to oxidized glutathione within cells is often used as a marker of oxidative stress^42^. NLRP3 inflammasome activated caspase-1 promotes multiple pathways causing mitochondrial disassembly, resulting in dissipation of mitochondrial membrane potential, mitochondrial permeabilization and mitochondrial ROS production^43^. Therefore, we sought to determine the GSH:GSSG (Glutathione disulfide) ratio to check the oxidative stress induced by α-hemolysin in THP-1m. It was found that during HlyA stimulation GSH level was reduced (Fig. 5A) in order to tackle the oxidative stress and is oxidized to GSSG (Fig. 5B), thus leading to a low GSH:GSSG ratio (Fig. 5C). Upon inhibition of the K^+^ efflux by using glibenclamide and a high K^+^ containing medium, the GSH:GSSG ratio was significantly improved (Fig. 5C).

### HlyA promotes mitochondrial biogenesis in THP-1m upon stimulation

**Figure 6:**
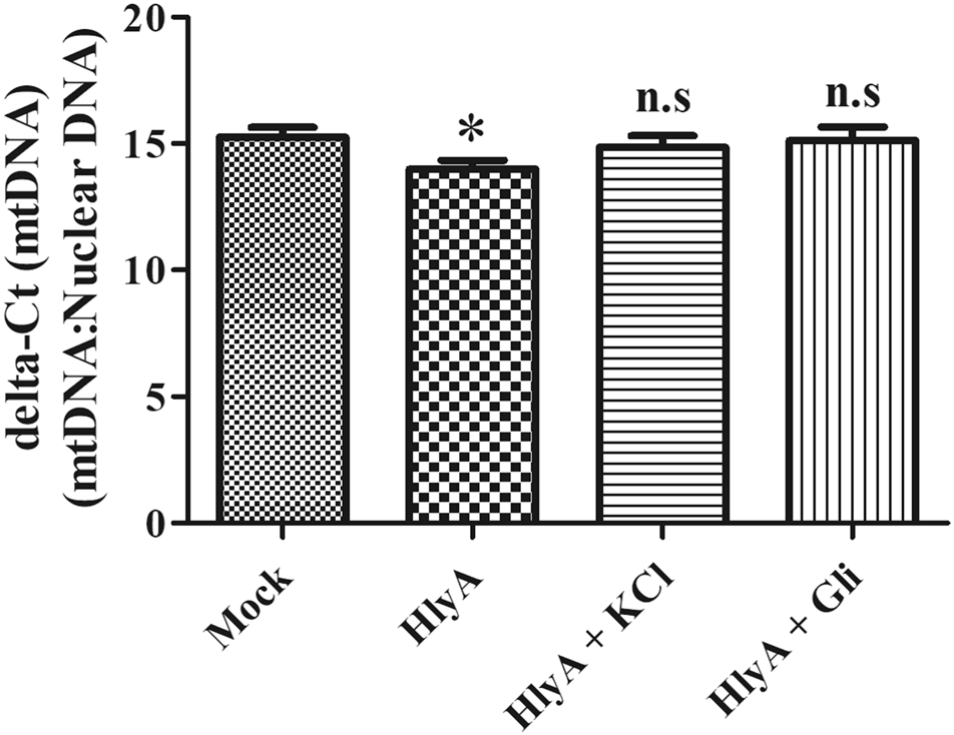
Mitochondrial Biogenesis was increased in THP-1m cells upon stimulation with HlyA. mtDNA copy number was quantified through quantitative real-time PCR in THP-1m cells. THP-1m cells were stimulated with HlyA for 2 h and prior to stimulation of THP-1m, cells were treated with 140 mM of potassium chloride (KCl) and 100 μM of glibenclamide (Gli) for 30 min as indicated. mtDNA copy numbers were normalized to the nuclear DNA copy number of 18S and represented as delta-Ct values. Lower delta-Ct indicates an increase in gene expression and vice versa. The graph shows a significant (*p = 0.01) increase in mtDNA only during HlyA stimulation, as compared to the mock and other stimulations of HlyA, where cells were pretreated with Gli and KCl. Results are representative of three biological and three technical replicates. n.s = non significant. Comparisons between multiple groups were made using one-way ANOVA with Bonferroni’s post test.

In various pathologies, it is evident that the increase in mitochondrial dysfunction is combated through mitochondrial biogenesis, in which dysfunctional mitochondria are replaced with healthy mitochondria to promote cell survival^44^. Therefore, we investigated the mitochondrial DNA copy number through quantitative real-time PCR. It was found that mtDNA copies were significantly (p = 0.0125) increased during HlyA stimulation of THP-1m, which was not seen on the inhibition of K^+^ efflux (Fig. 6), indicating a possible role of potassium efflux in the regulation of mitochondrial biogenesis.

### Quenching of mitochondrial ROS reduced IL-1β release in HlyA stimulated THP-1m

**Figure 7:**
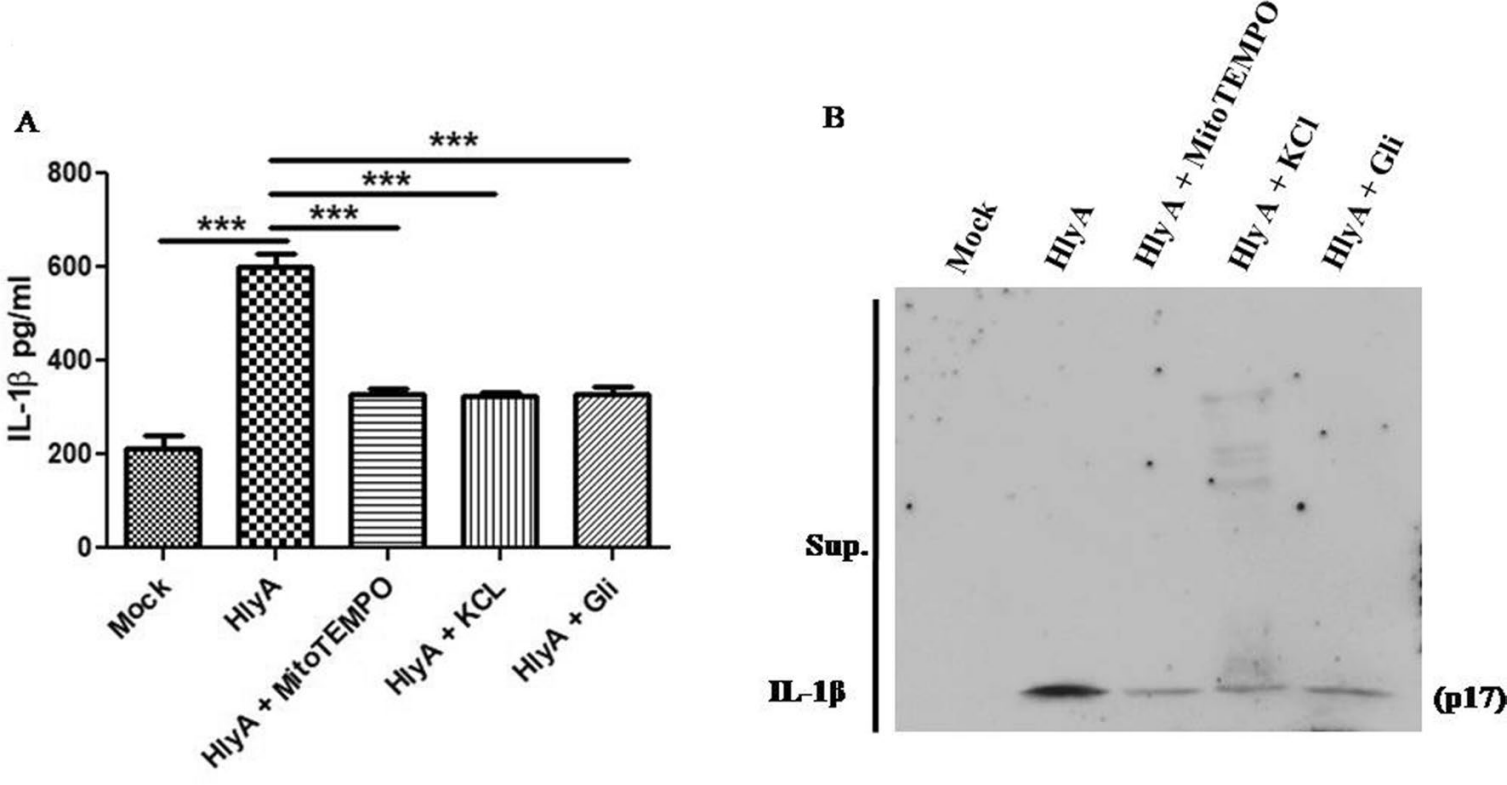
Cytokine IL-1β release during α-hemolysin stimulation along with mitoROS inhibition and blockage of potassium efflux. (**A**) ELISA was performed to assess IL-1β release in cell culture supernatants during HlyA stimulation, along with mitochondrial ROS inhibitor (MitoTEMPO 20 μM) and potassium efflux inhibition (140 mM KCl and Glibenclamide 100 μM). Data is represented as IL-1β concentration in pg/ml (Mean ± SEM). For statistical analysis, one-way ANOVA with Bonferroni’s test for multiple comparisons was used. P value is shown as ***p ≤ 0.001. (**B**) Immunoblot showing the cleaved form of IL-1β in acetone precipitated culture supernatants.

Zhou et al. demonstrated mitochondrial ROS as an essential mediator of NLRP3 inflammasome activation during stimulations, with known NLRP3 activators^40^. We investigated the role of mitochondrial ROS in NLRP3 activation by assessing the levels of IL-1β in cell culture medium (Supplementary Information 1), after stimulations. HlyA stimulation induced robust IL-1β release from THP-1m as compared to the mock (p ≤ 0.0001) (Fig. 7). Whereas, cells treated with MitoTEMPO along with HlyA stimulation showed a significant decrease in IL-1β release (p ≤ 0.0001), in comparison to the cells treated with HlyA alone (Fig. 7). These observations are similar to the outcomes of the study by Zhou et al.^40^. Additionally, Heid et al. observed the abrogation of IL-1β release by using MitoTEMPO in nigericin and ATP treated cells^45^. Our data also confirms the role of mitochondrial ROS in NLRP3 mediated IL-1β release during UPEC HlyA stimulation.

### UPEC α-hemolysin induced cell death is reversed by maintaining potassium homeostasis

**Figure 8:**
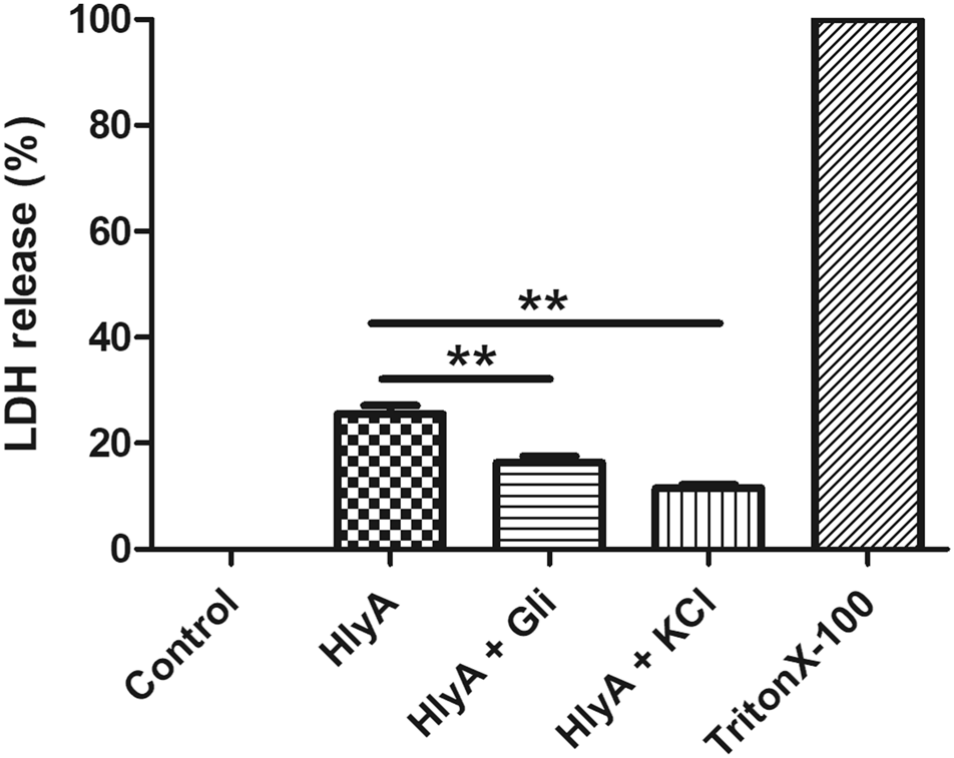
α-hemolysin induced cell death in THP-1 macrophages, reversed by inhibition of potassium efflux. LDH release assay was performed to evaluate the effect of α-hemolysin (HlyA) on THP-1m cell death. Additionally, the effect of glibenclamide and potassium chloride were assessed on hemolysin induced cell death of THP-1m. THP-1m pre-treated with glibenclamide (100 μM, 30 min prior to stimulation) and KCl (140 mM, prior 30 min to stimulation) were stimulated with α-hemolysin for 2 h. Data is represented as percentage LDH release (Mean ± SEM). Comparisons between multiple groups were made using one-way ANOVA with Bonferroni’s post test. P value is shown as ** ≤ 0.01.

Inflammasome activation leads to cytokine release and is accompanied by pyroptosis, leading to tissue damage. At lower concentrations, α-hemolysin was observed to induce apoptosis in host cells, including immune cells (neutrophils and T lymphocytes) and kidney cells (renal cells and bladder epithelial), ultimately causing exfoliation^17–19^. Here we assessed LDH release, in terms of percentage cytotoxicity in THP-1m cells upon HlyA stimulation for 2 h, with K^+^ concentration interventions. An α-hemolysin induced cell death in THP-1m (% cytotoxicity = 24.01 ± 0.08) was detected (Fig. 8). HlyA was reported to trigger K^+^ perturbances in the cells^34^. On the other hand, K^+^ concentration was reported to play an important role in assembly and activity of inflammasome^37–39^ and that the inflammasome activation can be prevented by blocking K^+^ efflux^37–39^. We have found that a higher concentration of K^+^ (140 mM) in cell culture medium reduces cell death (% cytotoxicity = 10.51 ± 0.30) (Fig. 8). Additionally, blockage of potassium efflux through glibenclamide also caused a decrease in cell death (% cytotoxicity = 16.38 ± 1.15) during HlyA stimulation (Fig. 8). Therefore, LDH assay results showed that upon α-hemolysin stimulation, cells undergo pyroptosis, which can be reversed through inhibition of potassium efflux.

## Discussion

α-hemolysin is an important virulence factor of UPEC, especially associated with severe upper tract pathogenesis of the urinary tract such as cystitis and pyelonephritis. Since it is associated with more than 50% of cases of severe UTI, therefore, it is thought to play an important role in the pathogenesis of UPEC^10,46^. Furthermore, *hlyA-*positive UPEC strains cause more tissue damage than *hlyA*-negative strains leading to severe clinical complications^11,46^. Earlier studies pointed towards the role of HlyA in inflammatory cell death^8,31,47^. We have characterized the role of HlyA in NLRP3 inflammasome activation by employing a recombinant purified active HlyA along with an inactive pro-hlyA, which was used to challenge human THP-1m. It was observed that HlyA promoted cytolytic activity and induced cleavage of IL-1β and caspase-1 in THP1m cells **(**Fig. 1A–C). We also demonstrated HlyA induced oligomerization of NLRP3 inflammasome (Fig. 1D). It is evident that HlyA induces deubiquitination of NLPR3, which is required for NLRP3 to be activated and initiate a pro-inflammatory response, in the form of IL-1β release (Fig. 1E). Craven et al. observed that NLRP3 dependent IL-1β release was inhibited in cells treated with 140 mM KCl during *S. aureus* α-hemolysin stimulation in THP-1 cells^48^. We observed that UPEC α-hemolysin induced IL-1β release and NLRP3 inflammasome oligomerization was dependent on K^+^ efflux (Fig. 2A,B,D). Interestingly, deubiquitination of NLRP3 was also affected in a similar way (Fig. 2E). Furthermore, it was shown that NLRP3 co-localizes within mitochondria followed by HlyA stimulation and causes mitochondrial dysfunction.

The NLRP3 inflammasome can be activated by diverse stimuli, which shows the involvement of many upstream receptors sensing different ligands causing similar cellular events, leading to the activation of NLRP3 inflammasome^49^. Though the exact mechanism remains unknown, literature suggests that NLRP3 inflammasome assembly activation involves potassium efflux from the cells, production of mitochondrial ROS, deubiquitination of NLRP3 followed by translocation to mitochondria, which triggers the release of mitochondrial DNA or cardiolipin from mitochondria; finally cathepsin-B release from lysosome into cytoplasm, followed by pyroptosis^50^. NLRP3 inflammasome is activated in response to UPEC infection and IL-1β is released in a caspase-1 dependent manner^8,51^. Mutant studies showed that the HlyA-positive UPEC strain induces NLRP3 expression and caspase-1 activation in bladder epithelial cells^8^. Further overexpression of HlyA in UPEC led to increased inflammasome activation and IL-1β secretion in the mouse, inducing inflammatory cell death in urothelial cells^31^. We observed that recombinant purified active HlyA induces caspase-1 activation and IL-1β maturation in THP-1m. In addition, we have also seen that HlyA induces deubiquitination of NLRP3 and its further assembly with ASC and pro-caspase-1. These results indicate that HlyA is one of the virulence factors of UPEC, which leads to the activation of NLRP3 inflammasome. To understand the mechanism of NLRP3 inflammasome activation during HlyA stimulation, we further looked into the effect of high extracellular K^+^ and K^+^ channel inhibitors (glibenclamide).

Potassium ion efflux is a characteristic of apoptotic cells and leads to caspase activation^52,53^; inhibiting K^+^ efflux delayed apoptosis through interference in cytochrome c release^52,54–56^, which implicated the association of K^+^ efflux with mitochondrial membrane integrity^56^. Potassium concentration in cells was shown to impact the health and functionality of cells; changes in intracellular K^+^ concentration have an effect on cell survival, mitochondrial health and immune response of neutrophils against bacteria^56–58^. Besides, HlyA was shown to alter signalling of host cells and affect viability and function of effector phagocytes^19,59–61^. Moreover, HlyA was reported to trigger K^+^ perturbances in the cells^34^. K^+^ concentration plays an important role in assembly and activity of inflammasome; inflammasome activation can be prevented by blocking K^+^ efflux^37–39^. We found that the maturation of both caspase-1 and IL-1β was inhibited by preventing K^+^ efflux using K^+^ ion channel inhibitor (glibenclamide) and high K^+^ concentration in the media (Fig. 2A–C). Similarly, Planillo et al. found that intracellular concentration of K^+^ regulates NLRP3 mediated caspase-1 activation during various stimulations with PFTs^38^. Moreover, inhibition of K^+^ efflux during HlyA stimulation could have led to the ubiquitination of NLRP3, which further impeded its oligomerization and activation (Fig. 2D,E). Therefore, we may speculate that deubiquitination, oligomerization and activation of NLRP3 depend on K^+^ efflux during HlyA stimulation. It would be interesting to elucidate further, that how K^+^ efflux regulates NLRP3 inflammasome activation or which biomolecules are modified during this process in order to regulate its activation. Almost all PFTs have been reported to work via K^+^ efflux mechanism for NLRP3 activation^62^. *E. coli* α-hemolysin induced cell death was decreased in THP-1 monocytes on inhibition of P2X7R receptor, known to be responsible for facilitation of K^+^ efflux during pore formation^63^. Whereas *S. aureus* α-hemolysin induced IL-1β release was found to be independent of P2X7R, but again dependent on intracellular K^+^ concentration^64^.

Despite energy production, mitochondria play an important role in cell death, innate immune response, cell metabolism and signaling by regulating the production of ROS. Therefore, mitochondria dysfunction has been observed during many diseases such as cancer, diabetes and infections, where pathogens specifically target mitochondria to gain hold of the cells and avoid cell death^65,66^. Bacterial virulence factors, mainly toxins, induce dysfunction in mitochondria to avoid apoptosis and phagocytosis to multiply inside the cells^65,67^. Earlier reports showed bacterial PFTs impede mitochondrial function by disturbing mitochondrial membrane potential and leakage of cytochrome c, inducing either apoptosis or necrosis^68,69^. Similarly, UPEC mediates programmed necrosis by impairing mitochondrial function. The UPEC HlyA, a PFT, causes mitochondrial fragmentation and loss of mitochondrial membrane potential, which finally results in mitochondrial dysfunction in sertoli cells of mice in in-vitro^28^. Besides mitochondrial dysfunction, UPEC causes permanent loss of plasma membrane integrity of sertoli cells, allowing the release of DAMPs, which cause activation of testicular macrophages by secretion of pro-inflammatory cytokines in-vitro^28^. We have also observed that stimulation with HlyA disturbed mitochondrial membrane potential and led to the depolarization of mitochondria in THP-1m (Fig. 4). In addition, NLRP3 was found to be co-localized within mitochondria, during stimulation of THP-1m with HlyA (Fig. 3). Activation of inflammasome was seen in cases where mitochondrial activity was disrupted. When the VDAC1 of mitochondria was inhibited, it resulted in NLRP3 inflammasome activation, clearly indicating the association with mitochondrial dysfunction^40^. We observed that blockage of K^+^ efflux (via glibenclamide or 140 mM KCl) prevented HlyA induced mitochondrial depolarization in THP-1m (Fig. 4), and this blockage is also reported to be associated with inhibition of NLRP3 inflammasome activation^37,39^. Therefore, we may interpret that UPEC HlyA induced disruption of mitochondrial membrane potential is dependent on K^+^ efflux and is also associated with NLRP3 inflammasome activation.

Studies have shown the importance of ROS, especially mitochondrial ROS, for activation of NLRP3 inflammasome^40^, but the role of ROS in NLRP3 inflammasome activation has not been well elucidated. Earlier, it was thought that ROS produced by NOX is important for NLRP3 activation^70^. Other studies have shown that inhibiting ROS has no effect on activation of NLRP3 and it just blocks priming of macrophages, i.e. mRNA expression of pro-IL-1β and NLRP3^71^. Further, ROS has a very short life span and acts only in the vicinity as a messenger^72^. Therefore, it was concluded that NLRP3 should be co-localized with mitochondria for efficient activation by ROS produced. We observed that NLRP3 is co-localized with mitochondria in THP-1m, when they were challenged with HlyA (Fig. 3). Zhou et al. also observed that in the presence of NLRP3 inflammasome activators, NLRP3 get colocalized with mitochondria and mitochondria-associated markers, where it can sense and regulate mitochondrial activity^40^. Mitochondrial ROS is an essential mediator of NLRP3 inflammasome activation during stimulations with known NLRP3 activators^40^. Similarly, Heid et al. saw ablation of IL-1β release in macrophages by quenching mitochondrial ROS via MitoTEMPO during stimulation with nigericin and ATP^45^. In contrast, Planillo et al. found role of intracellular K^+^ in NLRP3 activation, but they failed to observe any significant effect of mitochondrial dysfunction on IL-1β release^38^. Whereas we have found significant reduction in IL-1β release upon inhibition of Mitochondrial ROS (via MitoTEMPO) and potassium efflux during HlyA stimulation (Fig. 7). This shows that intracellular K^+^ and mitochondrial health regulate IL-1β secretion during a UPEC HlyA stimulation. Besides, heme is known to be associated with pathogenesis of hemolytic diseases, mainly sepsis, as it induces inflammation during infection via an unknown mechanism^73,74^. However, it was shown that blocking the oxidative effect of heme can save the tissue from death in case of sepsis^73,75^. Recently, heme was shown to be associated with NLRP3 inflammasome activation in a mitochondrial ROS (mitoROS) dependent manner, as blocking mitoROS avoided inflammasome activation by heme^76^. Therefore, we may interpret that mitoROS may be an important factor which helps in NLRP3 co-localization with mitochondria. It would be interesting to know how NLRP3 interacts with mitochondria and what could be the impacts of an association between NLRP3 and mitochondria.

To further assess the mitochondrial health upon HlyA stimulation, we measured the reduced/oxidized glutathione levels. The modulation of the redox microenvironment is an important regulator of immune cell activation and proliferation. Reduced glutathione (GSH) is considered to be one of the most important scavengers of ROS and its ratio with oxidized glutathione (GSSG) may be used as a marker of oxidative stress^77^. Our results showed that HlyA increased the oxidative stress in THP-1m, whereas inhibition of potassium efflux during HlyA stimulation improved GSH:GSSG ratio significantly (Fig. 5). It is well established that mitochondrial dysfunction is associated with many chronic inflammatory diseases and plays an important role in the pathogenesis of many disorders^78^. Mutation of genes involved in mitophagy has been found in diseases, such as Parkinson’s and Crohn’s disease^79^. Furthermore, a defect in mitophagy has been associated with increased production of IL-1β, a marker of inflammasome activation^80^. Mitophagy is also a cell defense mechanism to remove damaged mitochondria, which might block inflammasomes by inhibiting the activation of NLRP3 inflammasome^81^. Autophagy works through the removal of damaged mitochondria, a source of ROS, thus inhibiting the activation of the NLRP3 inflammasome. Blocked mitophagy caused increased activation of the NLRP3 inflammasome^40^. In addition, the removal of the damaged mitochondria induces the formation of new mitochondria. Recent studies have shown that mitochondrial biogenesis and mitophagy are coupled; for example, parkin regulates both mitophagy and mitochondrial biogenesis^82,83^. HlyA has been shown to induce the production of nitric oxide (NO) through inducible nitric-oxide-synthase (iNOS) pathway^84^ and NO induces PGC-1, a co-activator of mitochondrial replication, thus regulating mitochondrial biogenesis^85,86^. We observed that HlyA induces mitochondrial biogenesis, which might be a stress response by the THP-1m cells to remove dysfunctional mitochondria to control inflammation (Fig. 6). However, HlyA did not induce mitochondrial biogenesis during blockage of K^+^ efflux, which suggests that HlyA induced mitochondrial damage is protected by the inhibition of K^+^ efflux. Additionally, our findings suggest that the cell death induced by HlyA can be significantly reduced by inhibiting the activation of NLRP3 inflammasome, achieved by blocking K^+^ efflux through glibenclamide or by repleting intracellular K^+^ concentration (Fig. 8).

We may conclude that UPEC HlyA can induce the formation of the NLRP3 inflammasome by initiating deubiquitination of NLRP3, whereas proHlyA failed to do so. Therefore, we could suggest that the pore-forming property of UPEC α-hemolysin is necessary to initiate a pro-inflammatory response. In addition, results showed that HlyA induced activation of the NLRP3 inflammasome depends on the concentration of intracellular K^+^ and mitochondrial ROS during the stimulations of THP-1m. Similarly, K^+^ efflux due to HlyA stimulation resulted in the prevalence of dysfunctional mitochondria and localization of NLRP3 to the mitochondria for MitoROS sensing. It would be interesting to further investigate the contribution of mitochondrial dysfunction in detail during HlyA-induced NLRP3 inflammasome activation. During HlyA stimulation of THP-1m, an increase in mitochondrial biomass was observed, which could be a strategic stress response, but inhibition of K^+^ efflux neutralized this stress.

## Materials and methods

### Materials

Glibenclamide (Cat# G0639, Sigma-Aldrich, India) and Nigericin (Cat# 481990, Merck, India) were used in stimulation protocol. For RNA isolation and cDNA synthesis, TRI Reagent (Sigma cat # T9424; St Louis, Missouri, U.S.A) and RevertAid First Strand cDNA Synthesis Kit (Thermo Scientific Cat# K1622; Waltham, Massachusetts, U.S.A) were used, respectively. Protein estimation was done using a bicinchoninic acid (BCA) kit (Cat# 71285, Merck, India). Antibodies used were anti-NLRP3 antibody (Cat# NBP1-77080, Novus biological, USA), anti-Ubiquitin antibody (Cat# P497, Biolegend, California, USA), anti-ASC (Cat# NBP1-78977, Novus biological, USA), anti-VDAC antibody (Cat# 820701, Biolegend, California, USA), anti-Tubulin antibody (Cat# ab6046, Abcam, Cambridge, USA), anti-IL-1β antibody (ab2105, Abcam, Cambridge, USA), anti-Caspase-1 antibody (Cat# NBP1-45433, Novus biological, USA) and anti-GAPDH antibody (Cat# SC47724, Santa Cruz, California, USA). HRP-labelled anti-mouse (Cat# SC2005, Santa Cruz, California, USA) and anti-rabbit secondary antibody (Cat# ab6721, Abcam, Cambridge, USA) were used for immunoblotting. Clarity Western ECL substrate (Cat# 1705060, Bio-Rad, California, USA) was used for chemiluminescence. For confocal microscopy, Mitotracker Red CMXRos (Cat# M7512, Thermo Fisher Scientific, USA), Alexa fluor 488 (Cat# A-11012, Thermo Fisher Scientific, USA), VECTASHIELD Antifade Mounting Medium (Cat# H-1000, Vector laboratories, USA) and JC-1 Mitochondrial Membrane Potential Probe (Cat# T3168, Thermo Fisher Scientific, USA) were used. For GSH and GSSG estimation O-Phthaladehyde (OPT) (Cat # 27329, SRL) and N-Ethylmaleimide (NEM) (Cat # 78503, SRL) were used. For enzyme-linked immunosorbent assay (ELISA) of IL-1β cytokine, Human IL-1β GENLISA ELISA kit (Cat# KB1063, Krishgen Biosystems) was used and MitoTEMPO (Cat# SML0737, Sigma-Aldrich, India) was used for mitoROS inhibition. For LDH release Assay Cayman LDH cytotoxicity assay kit (Cat # 10009172) was used.

### Toxin preparation

Acylated Active (HlyA) and inactive α-hemolysin (proHlyA) were recombinantly produced according to the previous method^29^. Recently, we have reported a simple method for the recombinant production of hexa-histidine tagged active and inactive α-hemolysin by cloning only *hlyA* and *hlyC* genes of operon *hlyCABD*^29^. Fatty acid acylation of HlyA at lysine 564 and 590 residue is an important step, which is performed by HlyC^12^. Therefore, we have cloned *hlyA* and *hlyC* simultaneously for the production of active α-hemolysin, while *hlyA* was cloned alone to produce inactive α-hemolysin (non-acylated). Over-expression was achieved with 1 mM isopropyl-1-thio-β-d-galactopyranoside (IPTG) at 18 °C for 6 h. Both active and inactive forms of HlyA were purified by a batch purification method as described earlier^29^. Protein was subjected to desalting and concentration through Amicon Ultra-0.5 ml centrifugal filters (Merck) for 50 kDa molecular weight cut off (MWCO). Protein was eluted in 1X PBS supplemented with 20 mM CaCl_2_. Purified active and inactive HlyA were quantified through SDS-PAGE and then further subjected to hemolysis assay. An insignificant amount of endotoxin contamination was found in the preparations when subjected to LAL Assay to produce any synergistic effect^29^. Therefore, in further experiments, wherever HlyA was required, a batch of HlyA showing more than 90% of activity was used.

### Cell culture

THP-1 cells (cat # TIB-202, ATCC, Manassas, VA) were cultured in RPMI-1640 medium supplemented with 10% heat inactivated fetal bovine serum, 2 mM l-glutamine, 1 mM sodium pyruvate and 10 mM HEPES (cat#15630080, Life Technologies, Carlsbad, CA). THP-1 cells were differentiated into macrophage-like cells (THP-1m) by culturing for 48 h in a medium containing 25 nM Phorbol 12-myristate 13-acetate (PMA) (Sigma-Aldrich, St Louis, MO) and followed by 24 h of rest before any stimulation^87^.

### Stimulation protocol

THP-1m were incubated in RPMI 1,640 medium with HlyA and proHlyA along with control (mock/without any stimulation) for 2 h^27^; 200 ng of HlyA and proHlyA was used for stimulations per million of THP-1m in 1 ml of medium^27^. Nigericin was used at 15 μM concentration for 30 min for stimulations^40^. For experiments involving K^+^ concentration interventions, 140 mM potassium chloride (KCl) was added to the medium 30 min prior to the stimulation with HlyA^39,88^. Additionally, THP-1m cells were treated with 100 μM of glibenclamide (Gli) for 30 min prior to incubation with HlyA wherever indicated^39,89^.

### Lysate preparation of THP-1 cells

Cytoplasmic extract was prepared by lysing cells using cold Radio-immunoprecipitation assay (RIPA) lysis buffer (150 mM NaCl, 1% Nonidet P-40, 0.5% Sodium deoxycholate, 0.1% Sodium dodecyl sulphate and 25 mM Tris)^90^ (pH 7.4) supplemented with protease inhibitor cocktail for 15 min on ice. Homogenous lysis was achieved by passing cell suspension through a 28 gauge needle syringe (Dispovan, India) and then lysates were cleared by centrifugation at 13,000 rpm for 20 min. The supernatant was collected as cytoplasmic extract and stored at − 80 °C until further use.

### Western blotting

Protein was estimated using a BCA kit (Merck) according to the manufacturer’s instructions. 50 µg of protein was resolved on 15% or 8% SDS-PAGE gel (as required to detect desired molecular weight of protein) and subsequently transferred onto PVDF membrane (GE Healthcare Life Sciences), kept overnight at 25 V and at 4 °C. The various protein molecules were probed with specific primary antibodies (IL-1β, Caspase-1, NLRP3, Ubiquitin, ASC, VDAC-1, Tubulin and GAPDH) wherever required, followed by HRP-labelled anti-mouse and anti-rabbit secondary antibodies. The Clarity Western ECL substrate (Bio-Rad) was used to develop the blot by chemiluminescence. GAPDH and VDAC-1 were used as loading controls in immunoblotting for cytoplasmic extracts and mitochondrial fractions, respectively. Tubulin was immunoblotted to check for contamination of cytoplasmic content in mitochondrial fractions. Quantification was carried out using ImageJ software (NIH).

### Cytokine measurement

Differentiated THP-1m cells were stimulated with HlyA for 2 h. Before stimulation, cells were incubated in high K^+^ containing media and glibenclamide, as indicated earlier in the stimulation protocol. One group was treated with MitoTEMPO (20 µM for 1 h), to see the effect of mitochondrial ROS during HlyA stimulation on IL-1β release. The expression of cytokine IL-1β was estimated by ELISA using IL-1β ELISA kit (Cat# KB1063, Krishgen Biosystems). After incubations, the culture medium was harvested, filtered (0.2 μm filters) and ELISA was performed following the manufacturer’s instructions. Each experiment was performed three times and statistical analysis was done.

### Acetone precipitation of cell culture supernatant

For detecting IL-1β release through Western blotting, cell culture supernatants from various stimulations were subjected to acetone precipitation of proteins. Protein was precipitated by adding four volumes of chilled acetone to the sample (culture medium) followed by incubation at − 20 °C for 1 h. The mixture was then centrifuged at 13,000 rpm for 15 min and the pellet obtained was dissolved in 1% SDS supplemented with 1× protease inhibitor.

### Confocal microscopy

PMA treated THP-1 cells were seeded and differentiated into THP-1m in 8-well chamber slides at a concentration of 10^5^ cells/well, as mentioned earlier. THP-1m were stimulated with 200 ng of HlyA for 2 h. After 2 h, THP-1m were washed thrice with 1× PBS and incubated with 200 nM Mitotracker Red CMXRos for 45 min at 37 °C, followed by washing thrice with PBS for 5 min each. The cells were washed twice with 1× PBS and subsequently fixed with 4% paraformaldehyde (pH 7.4) for 30 min at 37 °C. Further, the cells were permeabilized using 0.15% Triton X-100 in 1× PBS for 10 min at room temperature in darkness. Thereafter, the cells were washed thrice with PBS followed by blocking with 1% BSA in PBST for 30 min at room temperature in darkness. The cells were subsequently incubated with anti-NLRP3 antibody at a concentration of 20 μg/ml in 1% BSA in PBS overnight at 4 °C in darkness. The cells were again washed thrice with 1× PBS for 5 min and incubated with a secondary antibody Alexa fluor 488 at a concentration of 2 μg/ml in 1× PBS with 1% BSA for 2 h at room temperature in darkness. Subsequently, the cells were washed thrice with 1× PBS for 5 min and incubated with DAPI at 300 nM concentration in 1× PBS for 5 min, followed by washing thrice in 1× PBS. The cells were mounted with VECTASHIELD Antifade Mounting Medium. Confocal imaging was performed with a Nikon A1 laser scan confocal microscope with Plan Apooptics, equipped with an argon laser. Data was analyzed using the NIS Elements Advanced Research software.

### Mitochondria isolation

Mitochondria isolation from THP-1m cells was performed using a method of Clayton et al.^91^ with minor modifications. THP-1m cells were seeded in 75 cm^2^ flasks and differentiated into THP-1m, as mentioned previously in the cell culture section of methods. Two flasks per treatment were used further for mitochondria isolation (~ 2 × 10^7^ cells). After harvesting cells, 9 ml of ice-cold RSB hypotonic buffer (10 mM NaCl, 1.5 mM MgCl_2_ and 10 mM Tris–HCl [pH 7.5]) was added to resuspend the pellet. The suspended cells were thereafter transferred to a 15 ml dounce homogenizer and kept on ice for 10–15 min. The cells were then broken mechanically by using a dounce homogenizer with a tight fitting Teflon pestle. Five cycles of 10–15 strokes, followed by a rest of 1 min on ice, were used to break open the cells. Immediately thereafter, 6 ml of 2.5× MS homogenization buffer (525 mM mannitol, 175 mM sucrose, 12.5 mM Tris–HCl [pH 7.5] and 2.5 mM EDTA [pH 7.5]) was added. The mixture was immediately invert-mixed 6–8 times to maintain tonicity and to prevent agglutination of organelles by sealing the mouth of the homogenizer with parafilm; the homogenate was then transferred to a 50 ml centrifuge tube. The volume of homogenate was raised up to 20 ml by adding 1× MS homogenization buffer (210 mM mannitol, 70 mM sucrose, 5 mM Tris–HCl [pH 7.5] and 1 mM EDTA [pH 7.5]). The mixture was centrifuged at 1,500*g* for 5 min at 4 °C to separate nuclei, unbroken cells and larger membrane fragments. The supernatant was transferred and 15 ml of 1× MS homogenization buffer was added and centrifuged at 1,500*g* for 5 min at 4 °C to remove cytoplasmic and nuclear content. This step was repeated one more time. Supernatant was transferred to a fresh and sterile centrifuge tube and mitochondria were pelleted down by centrifuging the tube at 13,000*g* for 15 min at 4 °C. Afterward, the mitochondrial pellet was washed twice using 10 ml of 1× MS homogenization buffer. Ultimately, the mitochondrial pellet was used for immunoblotting. The pellet was dissolved in 50 μl of RIPA buffer supplemented with 1× protease inhibitor cocktail and 10 μl of mitochondrial fraction was used per lane to detect various proteins as indicated.

### Mitochondrial membrane potential assay

Differentiated THP-1m cells were stimulated with HlyA for 2 h. Prior to stimulation, the cells were incubated in high K^+^ containing media and glibenclamide as indicated earlier in the stimulation protocol. After all incubations and stimulations of THP-1m, the cells were washed with 1× PBS and stained for 30 min with 2 μM JC1 dye in RPMI-1640 medium. JC1, as a monomer, gives a green fluorescence at 529 nm. JC1 monomers move inside energized mitochondria depending on the membrane potential, resulting in the subsequent JC1 aggregates inside mitochondria, which gives a red fluorescence at 590 nm. Tecan Infinite 200 pro, a multi-well plate reader was used for red/green fluorescence analysis of JC1 dye assay. The experiment was performed three times and with two technical replicates.

### GSH:GSSG estimation

Glutathione estimation was performed using the method of Singh et al.^92^. For the measurement of GSH and GSSG, o-phthalaldehyde (OPT) has been used as a fluorescent reagent. OPT has an ability to react specifically with GSH at pH 8 and GSSG at pH 12. *N*-ethylmaleimide (NEM) has been used to prevent auto-oxidation of GSH during measurement of GSSG in the present protocol.

For the experiment, PMA treated THP-1 cells were seeded in the 6-well plate at a density of 1.5 × 10^6^ cells/well and the cells were stimulated with HlyA for 2 h, as mentioned in the stimulation protocol. For K^+^ concentration interventions, 140 mM potassium chloride (KCl) was added to the medium 30 min prior to the stimulation with HlyA^39,88^. Additionally, THP-1m cells were treated with 100 μM of glibenclamide (Gli) for 30 min prior to incubation with HlyA, wherever indicated^39,89^. Culture media was removed and washed with 1× PBS and then 1 ml of 0.1 M potassium phosphate buffer with EDTA (ice cold KPE buffer). The cells were scraped and collected in 1.5 ml centrifuge tubes. Then, centrifugation was done at 500*g* for 10 min at 4 °C and the supernatant was discarded; thereafter, 200 µl of KPE buffer with 1× protease inhibitor was added to dissolve the cell pellet. After sonication, lysed samples were centrifuged at 18,000*g* for 10 min at 4 °C and the resulting supernatants were collected in separate pre-cooled 1.5 ml centrifuge tubes (10 µl samples from each tube were collected in 0.5 ml tubes for protein estimation by BCA kit). After protein estimation, 10 µg of protein sample was precipitated. Initially, 80 µl of protein sample was mixed with 20 µl trichloroacetic acid (TCA) (50% stock concentration), vortexed and kept in ice for 10 min. The protein sample with TCA was centrifuged at 10,000*g* for 10 min at 4 °C; the supernatant was transferred into a fresh 1.5 ml centrifuge tube. For GSH estimation: 10 µl supernatant with equal volume of OPT (1 mg/ml) and 180 µl KPE buffer (pH-8) in a black 96-well plate was added. For GSSG estimation: 50 µl of the supernatant was transferred into a new centrifuge tube, 0.5 µl N-ethylmaleimide (stock concentration: 4 M) was added and mixed thoroughly, and incubated for 30 min at room temperature to inhibit GSH. 10 µl of this sample, 10 µl OPT and 180 µl 0.1 N NaOH (pH-12) were added in a black 96-well plate. The plate was incubated in the dark for 10 min. Fluorescence at λex: 355 nm and λem: 420 nm in a microplate reader was taken and the results were analyzed.

### Co-immunoprecipitation

To check endogenous levels of oligomerization and ubiquitination of NLRP3 during various stimulations, co-immunoprecipitation was performed as follows. Cytoplasmic extract of THP-1m after all stimulation protocols, which were subsequently stored at − 80 °C, were thawed on ice. Cytoplasmic extracts of THP-1m cells (500 μg) were incubated for each reaction with 1 μg of anti-ASC (Novus Biologicals) antibody and 20 μl of Recombinant Protein A-Sepharose 4B beads (Invitrogen) overnight at 4 °C on a rotary invert mixer. The next day, sepharose beads were washed thrice with RIPA lysis buffer followed by mixing sepharose beads with 2× SDS PAGE protein sample buffer (80 mM Tris HCl (pH6.8), 10% (v/v) Glycerol, 2% SDS, 238 mM β-Mercaptoethanol, 0.0006% (v/v) Bromophenol blue and 0.1 M dithiothreitol [freshly added])^93^. The samples were then boiled at 95 °C for 5 min and run on 8% resolving SDS-PAGE. To check endogenous levels of ASC in input lysates (cytoplasmic extract), 30 μg of input lysates were run on 15% SDS-PAGE. For immunoblotting, the protocol was followed as described earlier. Each experiment included three biological and two technical replicates.

### Mitochondrial copy number

Mitochondrial copy number was determined by quantitative real-time PCR (RT-qPCR). Total RNA was isolated from stimulated THP-1m by TRI reagent (Sigma) as per the manufacturer’s instructions and quantified by using Nanodrop (ND-1000). A 500 ng of total RNA after quantification was subjected to cDNA synthesis using cDNA synthesis kit (RevertAid First Strand cDNA Synthesis Kit (Thermo Scientific)) as per the manufacturer’s protocol. Each experiment included three biological and three technical replicates. ABI 7,300 Real-Time PCR machine was used for quantification of mitochondrial DNA (mtDNA) by using Mesa green PCR Master mix (SYBR) (Eurogentec). The Real-time qPCR reaction contained 7.5 µl of 2× Mesa green PCR Master mix, 1 µl cDNA, 1 µM of each primer and water to make a final volume of 15 µl. qPCR conditions were: 50 °C for 5 min, 95 °C for 10 min, 40× (95 °C for 15 s and 60 °C for 1 min). The primers used for *mtDNA*^94^ and human *18S rRNA*^95^ are given in Table 1. Human *18S rRNA* nuclear amplicon was used as a housekeeping gene for internal control (or reference gene). mtDNA copy number was normalized to amplification of an 18S nuclear amplicon and calculated as mentioned previously^96^. Values of mtDNA are expressed as delta-Ct ± SD, lower delta-Ct indicates higher copy number. Normalization was done according to the given formula; delta-Ct (mtDNA) = (Ct of mtDNA in treatments) – (Ct of 18S in treatments). Three independent experiments were performed and statistical analysis was done.

**Table 1:** List of primers used for quantitative real time PCR (RT-qPCR).

S. no	Gene	Primer sequence	Reference
1	*mtDNA*	Forward: 5′-CCCCAGCCATAACACAGTATCAAAC-3’ Reverse: 5′-GCCCAAAGAATCAGAACAGATGC-3’	^94^
2	*18S rRNA*	Forward: 5′-GTGGTGTTGAGGAAAGCAGACA-3′ Reverse: 5′-TGATCACACGTTCCACCTCATC-3′	^95^

### LDH release assay

For LDH release assay, 50,000 cells/well were seeded in 96-well clear plate and were allowed to differentiate as mentioned in the cell culture method. Differentiated THP-1m were stimulated with HlyA for 2 h; prior to stimulation, cells were incubated in high K^+^ containing media and glibenclamide, as indicated earlier in the stimulation protocol. The experiment was performed according to the manufacturer’s instruction. After 2 h, the plate was centrifuged at 400*g* for 10 min at room temperature. 100 μl of culture medium was transferred into another well and 100 μl of LDH reaction solution was added. The resulting solution was incubated at 37˚C with gentle shaking for 30 min and then the absorbance was taken at 490 nm. The experiment was performed thrice with two technical replicates.

### Statistical analysis

All data sets were analyzed via one-way ANOVA followed by Bonferroni’s post-hoc analysis using GraphPad software (GraphPad). P values ≤ 0.05 were considered as statistically significant.

## Supplementary information

Supplementary Information 1.

## References

[1] Foxman B (2010). The epidemiology of urinary tract infection. Nat. Rev. Urol..

[2] Ragnarsdóttir B, Lutay N, Grönberg-Hernandez J, Köves B, Svanborg C (2011). Genetics of innate immunity and UTI susceptibility. Nat. Rev. Urol..

[3] Hotchkiss RS, Karl IE (2003). The pathophysiology and treatment of sepsis. N. Engl. J. Med..

[4] Hill M, Drasar B (1975). The normal colonic bacterial flora. Gut.

[5] Ronald A (2003). The etiology of urinary tract infection: Traditional and emerging pathogens. Dis. Mon..

[6] Aboderin OA, Abdu A-R, Odetoyin BW, Lamikanra A (2009). Antimicrobial resistance in *Escherichia coli* strains from urinary tract infections. J. Natl Med. Assoc..

[7] Mao B-H (2012). Identification of Escherichia coli genes associated with urinary tract infections. J. Clin. Microbiol..

[8] Schaale K (2016). Strain-and host species-specific inflammasome activation, IL-1β release, and cell death in macrophages infected with uropathogenic *Escherichia coli*. Mucosal Immunol..

[9] Schiwon M (2014). Crosstalk between sentinel and helper macrophages permits neutrophil migration into infected uroepithelium. Cell.

[10] Bien J, Sokolova O, Bozko P (2012). Role of uropathogenic *Escherichia coli* virulence factors in development of urinary tract infection and kidney damage. Int. J. Nephrol..

[11] Johnson JR (1991). Virulence factors in Escherichia coli urinary tract infection. Clin. Microbiol. Rev..

[12] Issartel J-P, Koronakis V, Hughes C (1991). Activation of *Escherichia coli* prohaemolysin to the mature toxin by acyl carrier protein-dependent fatty acylation. Nature.

[13] Stanley P, Packman LC, Koronakis V, Hughes C (1994). Fatty acylation of two internal lysine residues required for the toxic activity of *Escherichia coli* hemolysin. Science.

[14] Laestadius Å, Richter-Dahlfors A, Aperia A (2002). Dual effects of *Escherichia coli* α-hemolysin on rat renal proximal tubule cells. Kidney Int..

[15] Keane WF, Welch R, Gekker G, Peterson PK (1987). Mechanism of *Escherichia coli* alpha-hemolysin-induced injury to isolated renal tubular cells. Am. J. Pathol..

[16] Cavalieri SJ, Bohach GA, Snyder I (1984). *Escherichia coli *alpha-hemolysin: Characteristics and probable role in pathogenicity. Microbiol. Rev..

[17] Smith YC, Grande KK, Rasmussen SB, O'Brien AD (2006). Novel three-dimensional organoid model for evaluation of the interaction of uropathogenic *Escherichia coli* with terminally differentiated human urothelial cells. Infect. Immun..

[18] Jonas D, Schultheis B, Klas C, Krammer P, Bhakdi S (1993). Cytocidal effects of *Escherichia coli *hemolysin on human T lymphocytes. Infect. Immun..

[19] Russo TA (2005). *E. coli* virulence factor hemolysin induces neutrophil apoptosis and necrosis/lysis in vitro and necrosis/lysis and lung injury in a rat pneumonia model. Am. J. Physiol. Lung Cell. Mol. Physiol..

[20] Uhlén P (2000). α-Haemolysin of uropathogenic *E. coli* induces Ca^2+^ oscillations in renal epithelial cells. Nature.

[21] Schroder K, Tschopp J (2010). The inflammasomes. Cell.

[22] Verma V, Dhanda RS, Møller NF, Yadav M (2016). Inflammasomes and their role in innate immunity of sexually transmitted infections. Front. Immunol..

[23] Waldhuber A (2016). Uropathogenic *Escherichia coli* strain CFT073 disrupts NLRP3 inflammasome activation. J. Clin. Investig..

[24] He Y, Hara H, Núñez G (2016). Mechanism and regulation of NLRP3 inflammasome activation. Trends Biochem. Sci..

[25] Jo EK, Kim JK, Shin DM, Sasakawa C (2016). Molecular mechanisms regulating NLRP3 inflammasome activation. Cell Mol. Immunol..

[26] Murthy AMV (2018). Regulation of hemolysin in uropathogenic *Escherichia coli* fine-tunes killing of human macrophages. Virulence.

[27] Bhakdi S, Muhly M, Korom S, Schmidt G (1990). Effects of *Escherichia coli* hemolysin on human monocytes. Cytocidal action and stimulation of interleukin 1 release. J. Clin. Investig..

[28] Lu Y (2018). Uropathogenic *Escherichia coli* virulence factor hemolysin A causes programmed cell necrosis by altering mitochondrial dynamics. FASEB J..

[29] Verma V (2019). Efficient production of endotoxin depleted bioactive α-hemolysin of uropathogenic *Escherichia coli*. Prep. Biochem. Biotechnol..

[30] Schwarz H, Schmittner M, Duschl A, Horejs-Hoeck J (2014). Residual endotoxin contaminations in recombinant proteins are sufficient to activate human CD1c+ dendritic cells. PLoS ONE.

[31] Nagamatsu K (2015). Dysregulation of *Escherichia coli* α-hemolysin expression alters the course of acute and persistent urinary tract infection. Proc. Natl. Acad. Sci..

[32] Demirel I (2018). Activation of the NLRP3 inflammasome pathway by uropathogenic *Escherichia coli *is virulence factor-dependent and influences colonization of bladder epithelial cells. Front. Cell. Infect. Microbiol..

[33] Skals M (2010). *Escherichia coli *α-hemolysin triggers shrinkage of erythrocytes via K(Ca)3.1 and TMEM16A channels with subsequent phosphatidylserine exposure. J. Biol. Chem..

[34] Kloft N (2009). Pore-forming toxins activate MAPK p38 by causing loss of cellular potassium. Biochem. Biophys. Res. Commun..

[35] Py BF, Kim M-S, Vakifahmetoglu-Norberg H, Yuan J (2013). Deubiquitination of NLRP3 by BRCC3 critically regulates inflammasome activity. Mol. Cell.

[36] Juliana C (2012). Non-transcriptional priming and deubiquitination regulate NLRP3 inflammasome activation. J. Biol. Chem..

[37] Petrilli V (2007). Activation of the NALP3 inflammasome is triggered by low intracellular potassium concentration. Cell Death Differ..

[38] Muñoz-Planillo R (2013). K+ efflux is the common trigger of NLRP3 inflammasome activation by bacterial toxins and particulate matter. Immunity.

[39] Arlehamn CSL, Pétrilli V, Gross O, Tschopp J, Evans TJ (2010). The role of potassium in inflammasome activation by bacteria. J. Biol. Chem..

[40] Zhou R, Yazdi AS, Menu P, Tschopp J (2011). A role for mitochondria in NLRP3 inflammasome activation. Nature.

[41] Pompella A, Visvikis A, Paolicchi A, De Tata V, Casini AF (2003). The changing faces of glutathione, a cellular protagonist. Biochem. Pharmacol..

[42] Townsend DM, Tew KD, Tapiero H (2003). The importance of glutathione in human disease. Biomed. Pharmacother..

[43] Yu J (2014). Inflammasome activation leads to Caspase-1–dependent mitochondrial damage and block of mitophagy. Proc. Natl. Acad. Sci..

[44] Golpich M (2017). Mitochondrial dysfunction and biogenesis in neurodegenerative diseases: Pathogenesis and treatment. CNS Neurosci. Ther..

[45] Heid ME (2013). Mitochondrial reactive oxygen species induces NLRP3-dependent lysosomal damage and inflammasome activation. J. Immunol..

[46] Marrs CF (2002). Variations in 10 putative uropathogen virulence genes among urinary, faecal and peri-urethral *Escherichia coli*. J. Med. Microbiol..

[47] Bhakdi S, Mackman N, Nicaud J, Holland I (1986). Escherichia coli hemolysin may damage target cell membranes by generating transmembrane pores. Infect. Immun..

[48] Craven RR (2009). *Staphylococcus aureus* α-hemolysin activates the NLRP3-inflammasome in human and mouse monocytic cells. PLoS ONE.

[49] Guo H, Callaway JB, Ting JP (2015). Inflammasomes: Mechanism of action, role in disease, and therapeutics. Nat. Med..

[50] Vanaja SK, Rathinam VA, Fitzgerald KA (2015). Mechanisms of inflammasome activation: Recent advances and novel insights. Trends Cell Biol..

[51] Verma V (2019). Involvement of NLRP3 and NLRC4 inflammasome in uropathogenic *E. coli* mediated urinary tract infections. Front. Microbiol..

[52] Bortner CD, Hughes FM, Cidlowski JA (1997). A primary role for K+ and Na^+^ efflux in the activation of apoptosis. J. Biol. Chem..

[53] Maeno E, Ishizaki Y, Kanaseki T, Hazama A, Okada Y (2000). Normotonic cell shrinkage because of disordered volume regulation is an early prerequisite to apoptosis. Proc. Natl. Acad. Sci..

[54] Colom LV (1998). Role of potassium channels in amyloid-induced cell death. J. Neurochem..

[55] Yu S, Yeh C-H, Strasser U, Tian M, Choi D (1999). NMDA receptor-mediated K+ efflux and neuronal apoptosis. Science.

[56] El Kebir D, József L, Khreiss T, Filep JG (2006). Inhibition of K+ efflux prevents mitochondrial dysfunction, and suppresses caspase-3-, apoptosis-inducing factor-, and endonuclease G-mediated constitutive apoptosis in human neutrophils. Cell. Signal..

[57] Krause K-H, Welsh MJ (1990). Voltage-dependent and Ca2(+)-activated ion channels in human neutrophils. J. Clin. Investig..

[58] Ahluwalia J (2004). The large-conductance Ca^2+^-activated K^+^ channel is essential for innate immunity. Nature.

[59] Cavalieri SJ, Snyder IS (1982). Effect of *Escherichia coli* alpha-hemolysin on human peripheral leukocyte viability in vitro. Infect. Immun..

[60] Wiles TJ, Bower JM, Redd MJ, Mulvey MA (2009). Use of zebrafish to probe the divergent virulence potentials and toxin requirements of extraintestinal pathogenic *Escherichia coli*. PLoS Pathog..

[61] Gadeberg OV, Hacker J, Ørskov I (1989). Role of α-hemolysin for the in vitro phagocytosis and intracellular killing of *Escherichia coli*. Zentralblatt für Bakteriologie.

[62] Greaney AJ, Leppla SH, Moayeri M (2015). Bacterial exotoxins and the inflammasome. Front. Immunol..

[63] Fagerberg SK, Jakobsen MR, Skals M, Praetorius HA (2016). Inhibition of P2X receptors protects human monocytes against damage by leukotoxin from Aggregatibacter actinomycetemcomitans and α-hemolysin from *Escherichia coli*. Infect. Immun..

[64] Muñoz-Planillo R, Franchi L, Miller LS, Núñez G (2009). A critical role for hemolysins and bacterial lipoproteins in *Staphylococcus aureus*-induced activation of the Nlrp3 inflammasome. J. Immunol..

[65] Jiang JH, Tong J, Gabriel K (2012). Hijacking mitochondria: Bacterial toxins that modulate mitochondrial function. IUBMB Life.

[66] Lobet E, Letesson J-J, Arnould T (2015). Mitochondria: A target for bacteria. Biochem. Pharmacol..

[67] Escoll P, Mondino S, Rolando M, Buchrieser C (2016). Targeting of host organelles by pathogenic bacteria: A sophisticated subversion strategy. Nat. Rev. Microbiol..

[68] Nougayrède JP, Donnenberg MS (2004). Enteropathogenic *Escherichia coli* EspF is targeted to mitochondria and is required to initiate the mitochondrial death pathway. Cell. Microbiol..

[69] Niu H, Kozjak-Pavlovic V, Rudel T, Rikihisa Y (2010). Anaplasma phagocytophilum Ats-1 is imported into host cell mitochondria and interferes with apoptosis induction. PLoS Pathog..

[70] Dostert C (2008). Innate immune activation through Nalp3 inflammasome sensing of asbestos and silica. Science.

[71] Bauernfeind FG (2009). Cutting edge: NF-κB activating pattern recognition and cytokine receptors license NLRP3 inflammasome activation by regulating NLRP3 expression. J. Immunol..

[72] Veal EA, Day AM, Morgan BA (2007). Hydrogen peroxide sensing and signaling. Mol. Cell.

[73] Larsen R (2010). A central role for free heme in the pathogenesis of severe sepsis. Sci. Transl. Med..

[74] Fernandez PL (2010). Heme amplifies the innate immune response to microbial molecules through spleen tyrosine kinase (Syk)-dependent reactive oxygen species generation. J. Biol. Chem..

[75] Fortes GB (2012). Heme induces programmed necrosis on macrophages through autocrine TNF and ROS production. Blood.

[76] Dutra FF (2014). Hemolysis-induced lethality involves inflammasome activation by heme. Proc. Natl. Acad. Sci..

[77] Zitka O (2012). Redox status expressed as GSH: GSSG ratio as a marker for oxidative stress in paediatric tumour patients. Oncol. Lett..

[78] Oliveira J (2010). Nature and cause of mitochondrial dysfunction in Huntington’s disease: Focusing on huntingtin and the striatum. J. Neurochem..

[79] Restivo NL, Srivastava MD, Schafer IA, Hoppel CL (2004). Mitochondrial dysfunction in a patient with crohn disease: Possible role in pathogenesis. J. Pediatr. Gastroenterol. Nutr..

[80] Saitoh T (2008). Loss of the autophagy protein Atg16L1 enhances endotoxin-induced IL-1β production. Nature.

[81] Goldman SJ, Taylor R, Zhang Y, Jin S (2010). Autophagy and the degradation of mitochondria. Mitochondrion.

[82] Scarffe LA, Stevens DA, Dawson VL, Dawson TM (2014). Parkin and PINK1: Much more than mitophagy. Trends Neurosci..

[83] Gaweda-Walerych K, Zekanowski C (2013). Integrated pathways of parkin control over mitochondrial maintenance-relevance to Parkinson's disease pathogenesis. Acta Neurobiol. Exp..

[84] Chen M (2004). Activation of extracellular signal-regulated kinase mediates apoptosis induced by uropathogenic *Escherichia coli* toxins via nitric oxide synthase: Protective role of heme oxygenase-1. J. Infect. Dis..

[85] Gutsaeva DR (2008). Transient hypoxia stimulates mitochondrial biogenesis in brain subcortex by a neuronal nitric oxide synthase-dependent mechanism. J. Neurosci..

[86] Reynolds CM (2009). Nitric oxide synthase-2 induction optimizes cardiac mitochondrial biogenesis after endotoxemia. Free Radic. Biol. Med..

[87] Lund ME, To J, O'Brien BA, Donnelly S (2016). The choice of phorbol 12-myristate 13-acetate differentiation protocol influences the response of THP-1 macrophages to a pro-inflammatory stimulus. J. Immunol. Methods.

[88] Gurcel L, Abrami L, Girardin S, Tschopp J, van der Goot FG (2006). Caspase-1 activation of lipid metabolic pathways in response to bacterial pore-forming toxins promotes cell survival. Cell.

[89] Muruve DA (2008). The inflammasome recognizes cytosolic microbial and host DNA and triggers an innate immune response. Nature.

[90] RIPA lysis buffer. *Cold Spring Harb. Protoc.*10.1101/pdb.rec101428 (2017).

[91] Clayton DA, Shadel GS (2014). Isolation of mitochondria from tissue culture cells. Cold Spring Harb. Protoc..

[92] Singh V, Gera R, Purohit MP, Patnaik S, Ghosh D (2017). Fluorometric Estimation of Glutathione in Cultured Microglial Cell Lysate. Bioprotocol.

[93] SDS–PAGE protein sample buffer (2×). *Cold Spring Harb. Protoc.*10.1101/pdb.rec073932 (2013).

[94] Mitchell T (2013). Dysfunctional mitochondrial bioenergetics and oxidative stress in Akita+/Ins2-derived β-cells. Am. J. Physiol. Endocrinol. Metab..

[95] Chandra S (2014). Association of angiotensin II type 1 receptor (A1166C) gene polymorphism and its increased expression in essential hypertension: A case-control study. PLoS ONE.

[96] Ginzinger DG (2002). Gene quantification using real-time quantitative PCR: An emerging technology hits the mainstream. Exp. Hematol..

