# Supplementary material for: α-Hemolysin of uropathogenic E. coli regulates NLRP3 inflammasome activation and mitochondrial dysfunction in THP-1 macrophages
Source: Sci Rep. 2020 Jul 28;10:12653. doi: 10.1038/s41598-020-69501-1 (PMC7387347; doi:10.1038/s41598-020-69501-1)
Supplement: Supplementary file 1 — Supplementary Information 1. [file 41598_2020_69501_MOESM1_ESM.pdf]

α-Hemolysin of Uropathogenic *E.coli* regulates NLRP3 inflammasome activation and mitochondrial dysfunction in THP-1 macrophages

Vivek Verma[1], Parveen Kumar[2], Surbhi Gupta[1], Sonal Yadav[1], Rakesh Singh Dhanda[3], Henrik Thorlacius[4]and Manisha Yadav[1*]

Figure 1

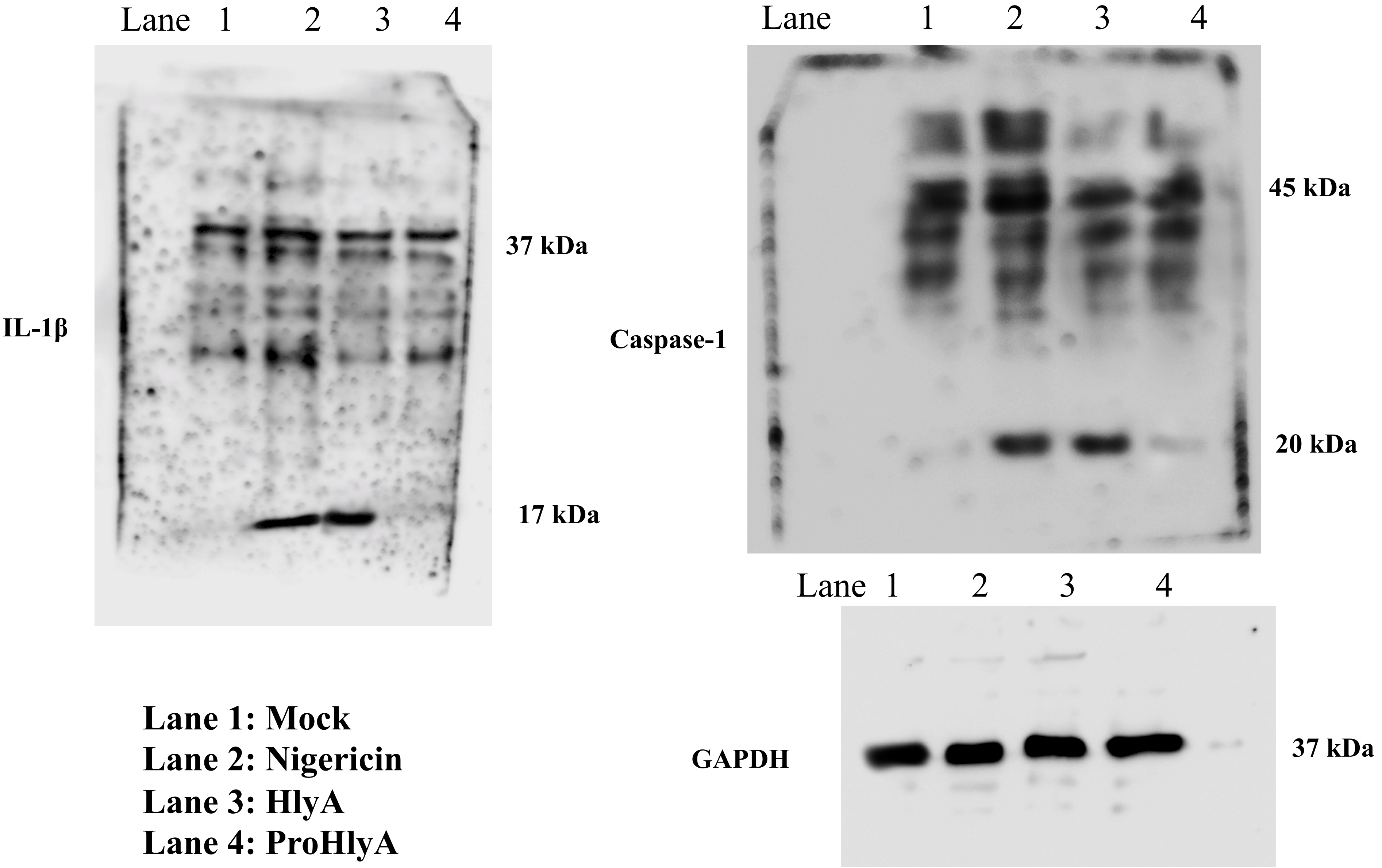

Lane 1: Mock
Lane 2: Nigericin
Lane 3: HlyA
Lane 4: ProHlyA

Cont.

Figure 1

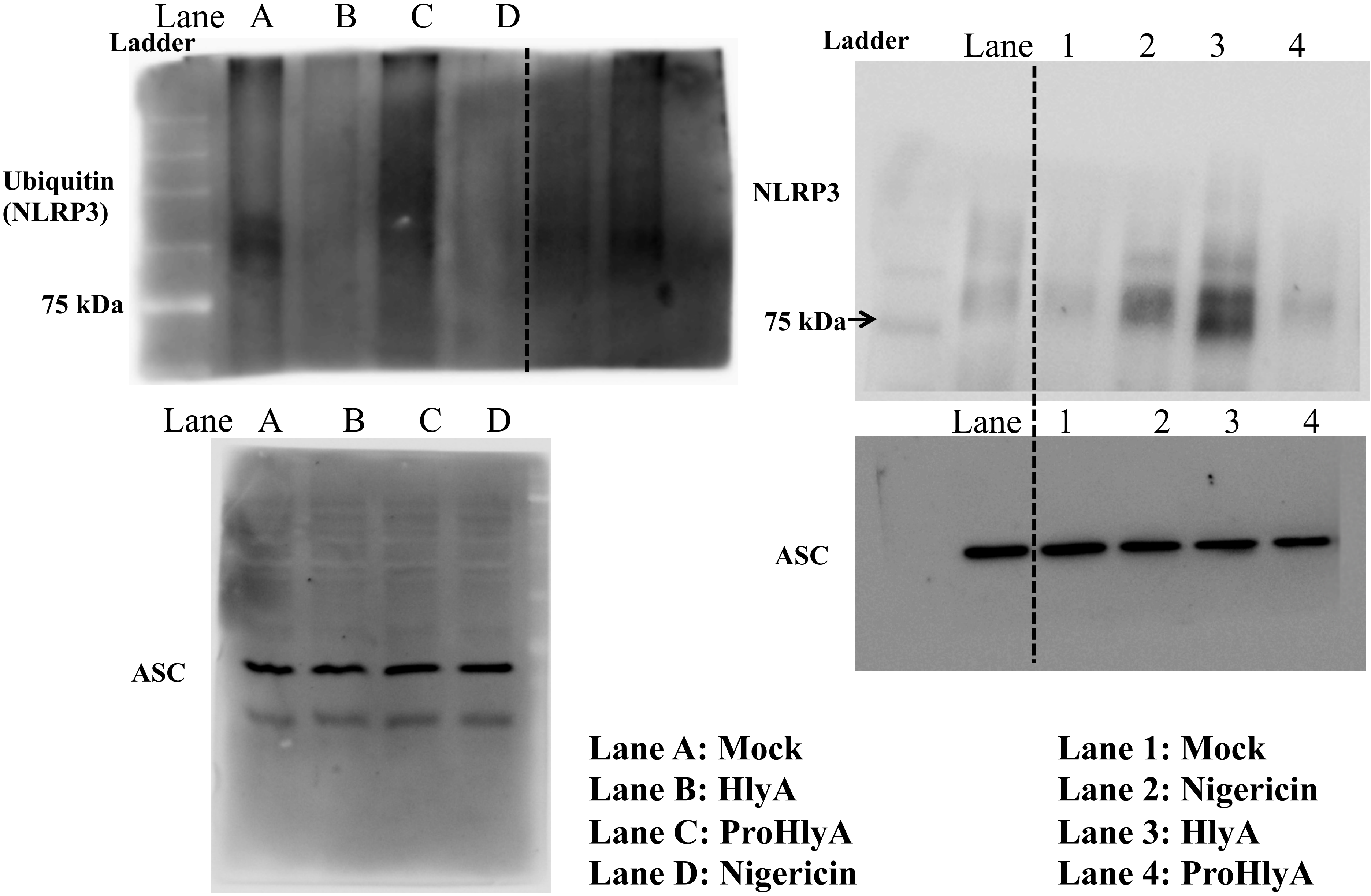

Lane A: Mock
Lane B: HlyA
Lane C: ProHlyA
Lane D: Nigericin

Lane 1: Mock
Lane 2: Nigericin
Lane 3: HlyA
Lane 4: ProHlyA

Figure 2

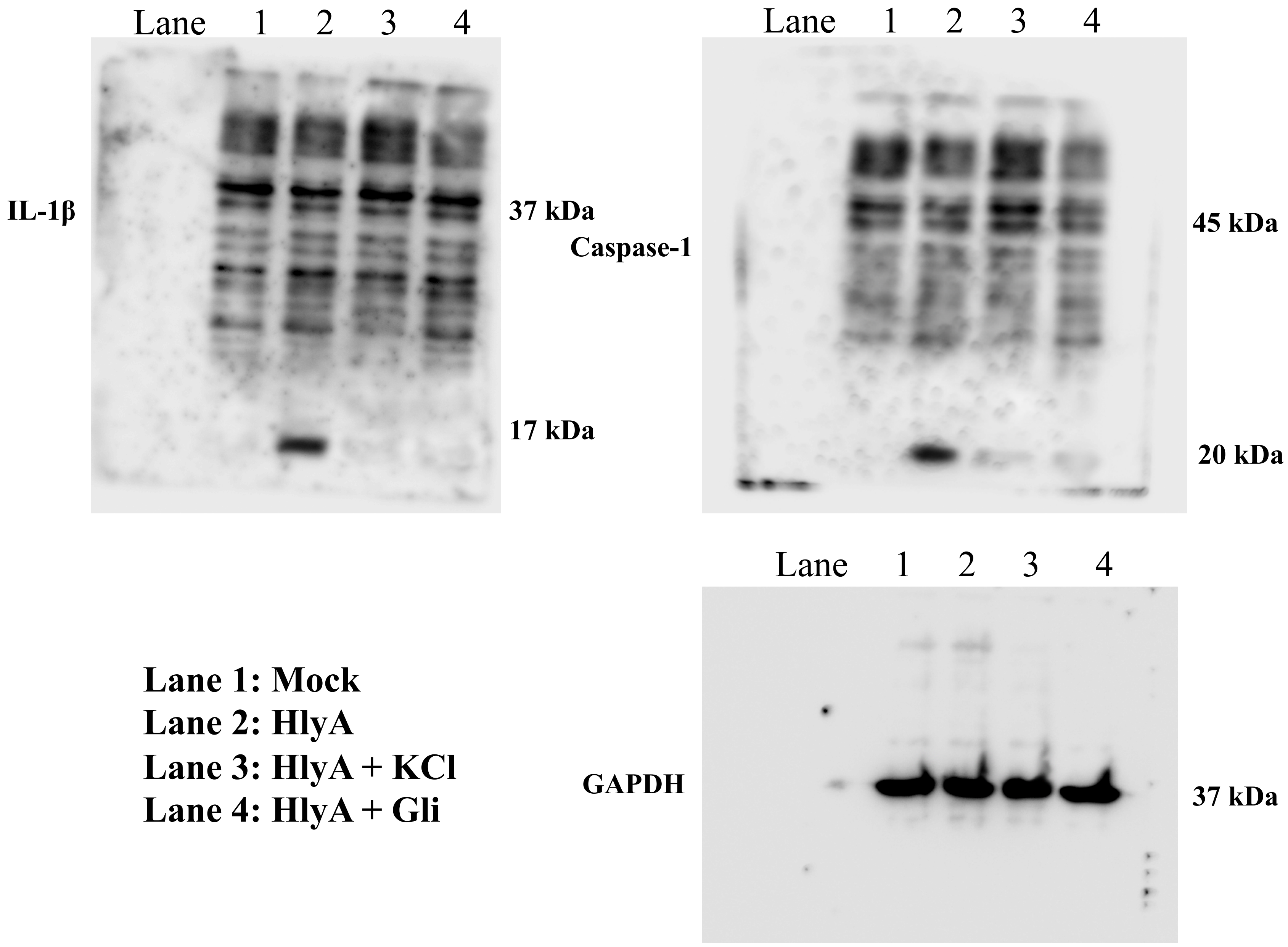

Cont.

Figure 2

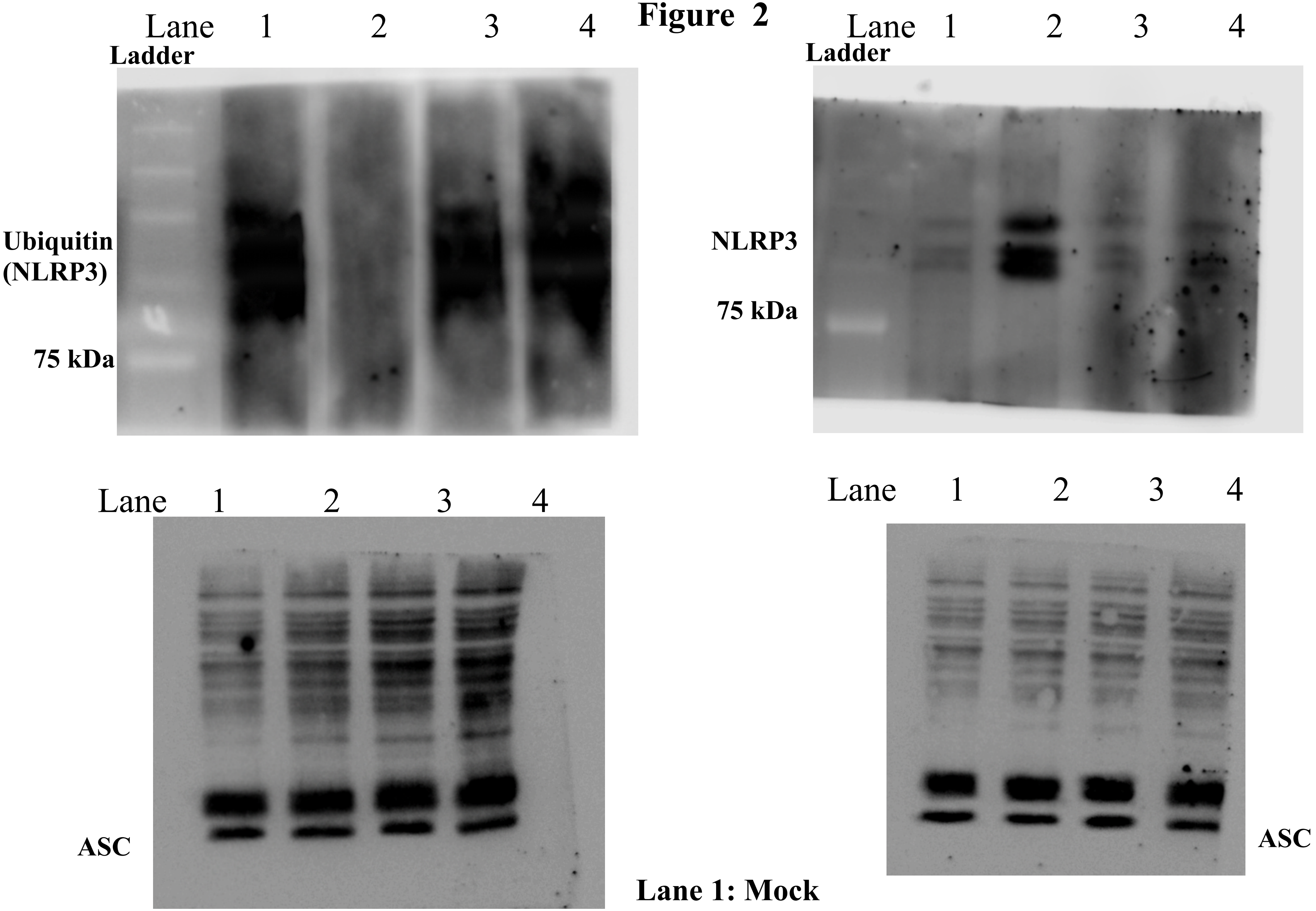

Lane 1: Mock
Lane 2: HlyA
Lane 3: HlyA + KCl
Lane 4: HlyA + Gli

Figure 3

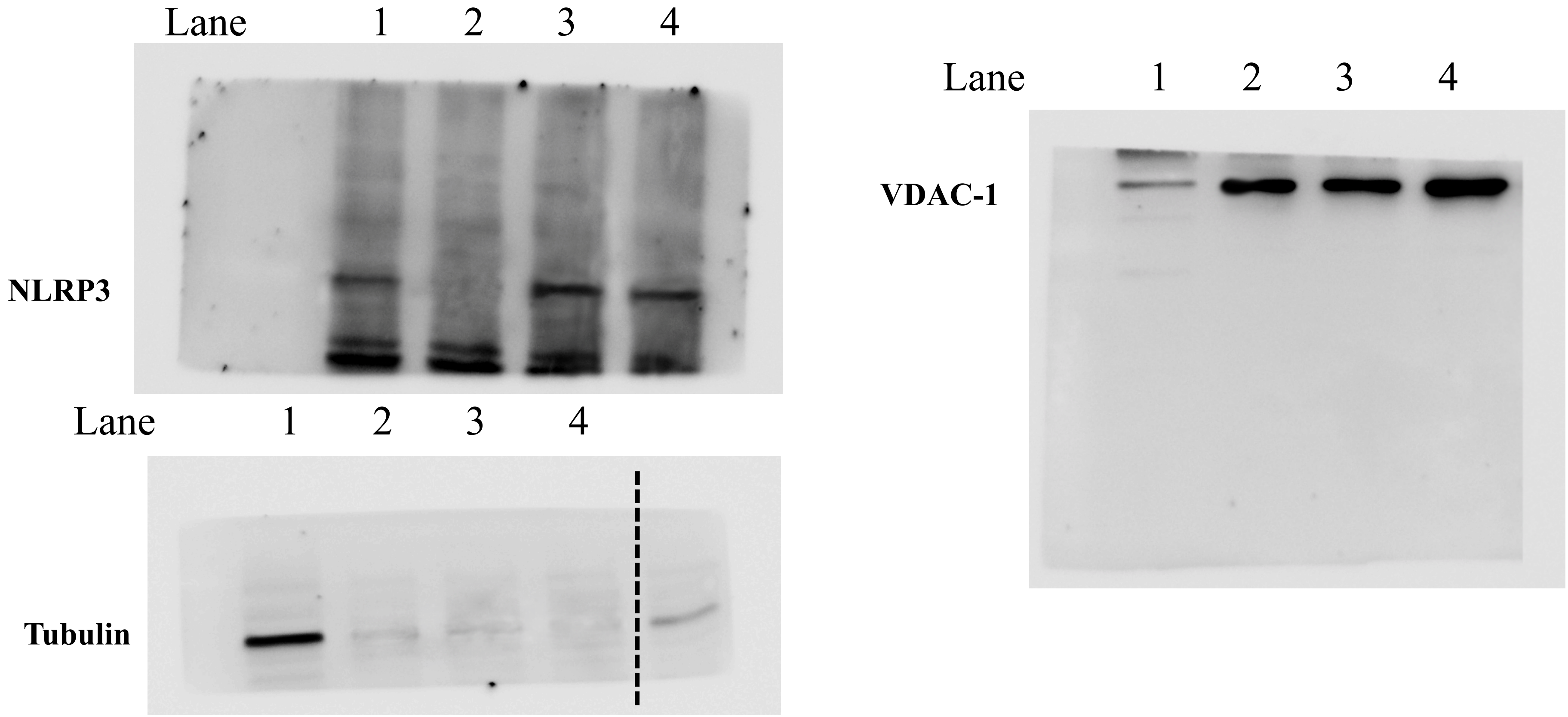

Lane 1: Mock (Cytoplasmic lysate)
Lane 2: Mock (Mitochondrial Lysate)
Lane 3: HlyA (Mitochondrial Lysate)
Lane 4: Nigericin (Mitochondrial Lysate)

Figure 7

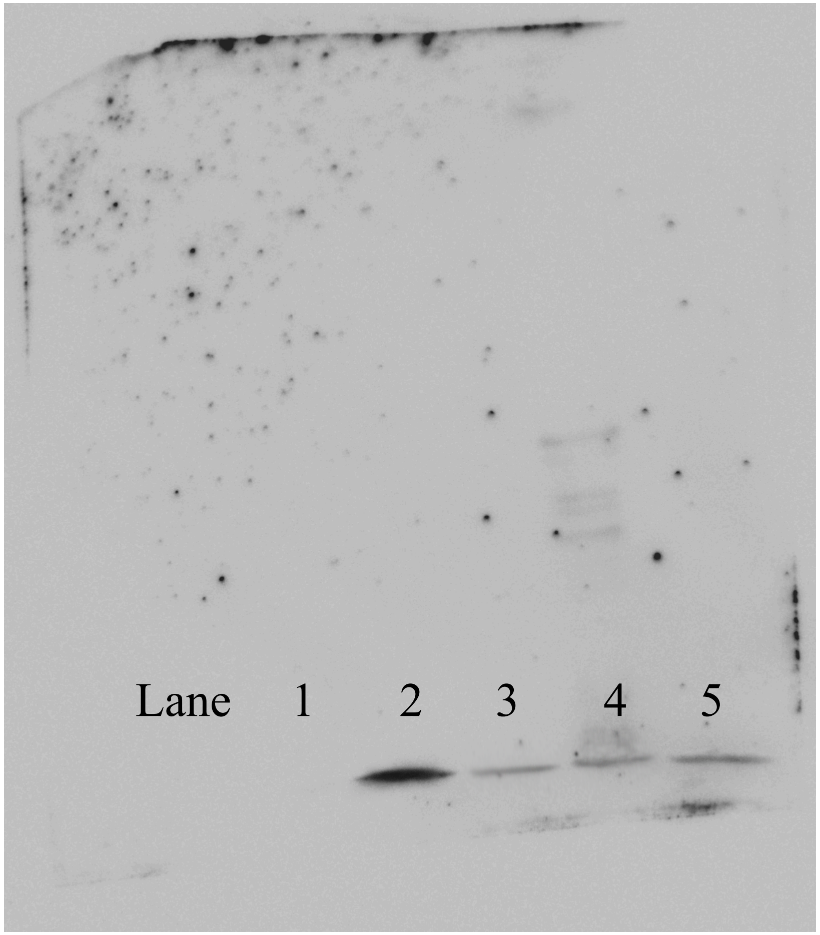

Lane 1: Mock
Lane 2: HlyA
Lane 3: HlyA + MitoTEMPO
Lane 4: HlyA + KCl
Lane 5: HlyA + Gli
